# Supplementary material for: Disassembly of chiral hydrogen-bonded frameworks into single-unit organometallic helices for enantioselective amyloid inhibition
Source: Nat Commun. 2025 Aug 27;16:8019. doi: 10.1038/s41467-025-63459-2 (PMC12391419; doi:10.1038/s41467-025-63459-2)
Supplement: Supplementary file 1 — Supplementary Information [file 41467_2025_63459_MOESM1_ESM.pdf]

Supplementary Information for

Disassembly of chiral hydrogen-bonded frameworks into single-unit organometallic helices for enantioselective amyloid inhibition

Yongli Ji[1]†, Caoyu Yang[2]†, Yutong Ye[1], Yin Zhang[3]*, Tingting Zhao[1], Shuyue Kong[1], Hongli Chen[1], Pai Liu[1], Zelong Zhao[1], Yilong Li[1], Jing Li[1], Ruixiao Ma[1], Zhiyong Ban[1], Kuo Yuan[4], Zhiyong Tang[2], Yi Liu[1]*, Meiting Zhao[5]* and Jun Guo[1]*

[1]State Key Laboratory of Advanced Separation Membrane Materials, School of Chemistry, Tiangong University, Tianjin 300387, P. R. China

[2]CAS Key Laboratory of Nanosystem and Hierarchical Fabrication, CAS Center for Excellence in Nanoscience, National Center for Nanoscience and Technology, Beijing 100190, P. R. China

[3]Department of Chemistry, University of North Texas, 1508 W Mulberry St, Denton, Texas 76201, USA

[4]Institute for New Energy Materials and Low Carbon Technologies, Tianjin University of Technology, Tianjin 300384, P. R. China

[5]Tianjin Key Laboratory of Molecular Optoelectronic Sciences, Department of Chemistry, Institute of Molecular Aggregation Science, Tianjin University, Tianjin 300072, P. R. China

†These authors contribute to this work equally.

*Corresponding author. Email: junguo@tiangong.edu.cn, mtzhao@tju.edu.cn, yiliuchem@whu.edu.cn, yin.zhang@unt.edu

Table of Contents

Supplementary figures Page 3

Supplementary tables Page 58

Supplementary references Page 80

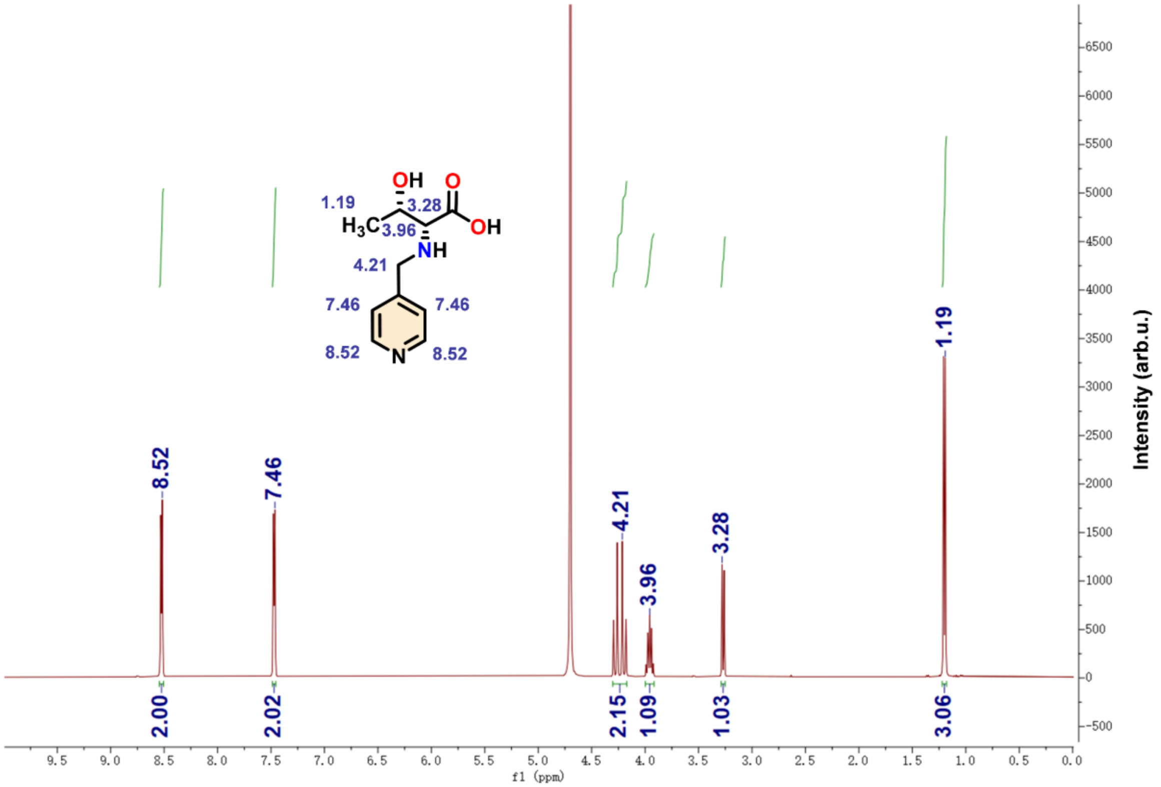

Supplementary Fig. 1. ^{1}H-NMR spectrum of *D*-Py-Thr. (The peak centered at 4.70 ppm is the residual solvent peak of D_2O). Peak assignments (ppm): -CH_3 (1.19, d, 3H), -HN-CH (3.28, d, 1H), -CH (3.96, m, 1H), -CH_2 (4.21 dd, 2H), py-H (7.46, d, 2H), py-H (8.52, d, 2H).

The ^{1}H-NMR spectrum demonstrates the successful synthesis of *D*-Py-Thr in high purity.

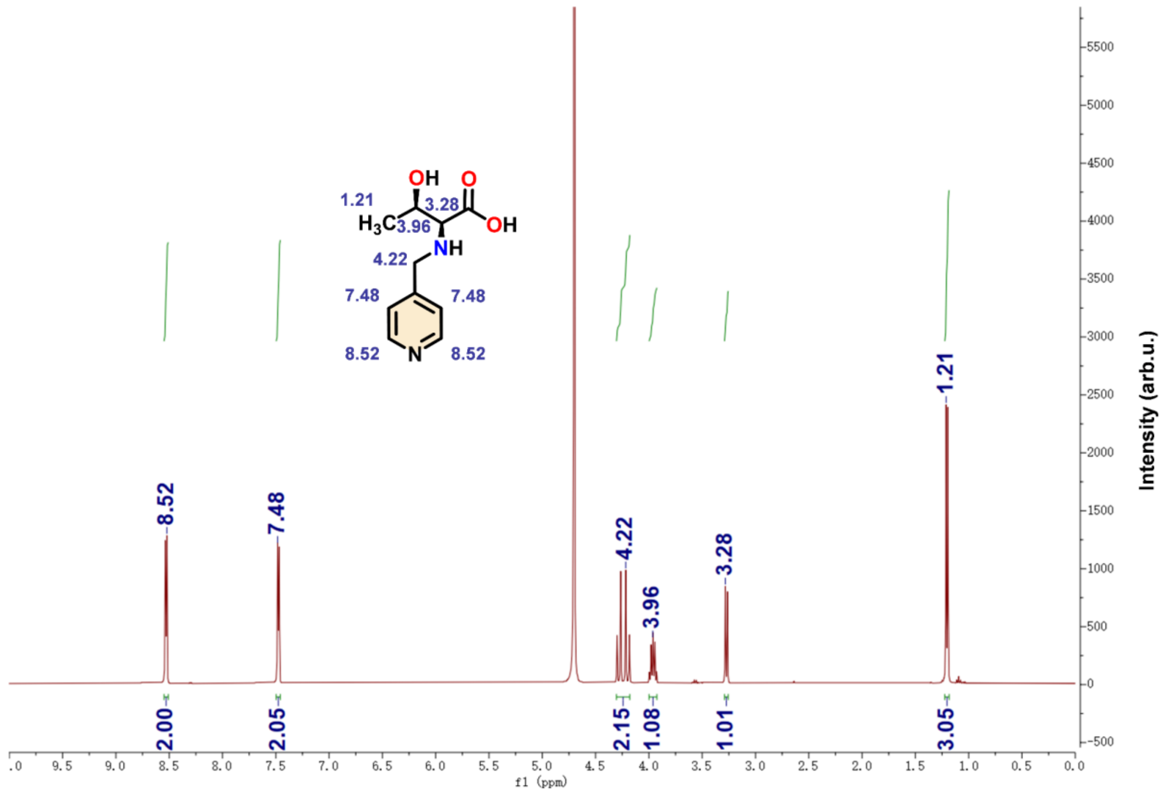

Supplementary Fig. 2. ^{1}H-NMR spectrum of *L*-Py-Thr. (The peak centered at 4.70 ppm is the residual solvent peak of D_2O). Peak assignments (ppm): -CH_3 (1.21, d, 3H), -HN-CH (3.28, d, 1H), -CH (3.96, m, 1H), -CH_2 (4.22 dd, 2H), py-H (7.48, d, 2H), py-H (8.52, d, 2H).

The ^{1}H-NMR spectrum demonstrates the successful synthesis of *L*-Py-Thr in high purity.

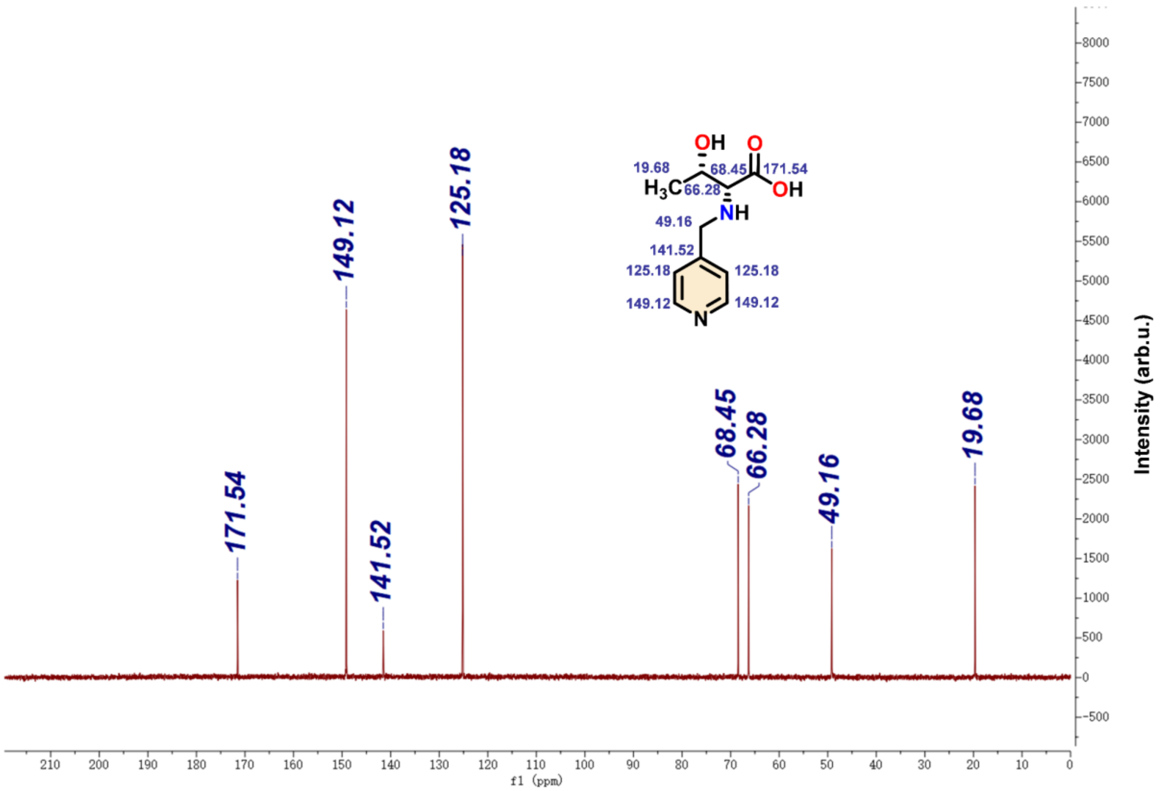

Supplementary Fig. 3. ^{13}C-NMR spectrum of *D*-Py-Thr. Peak assignments (ppm): -CH_3 (19.68), -CH_2 (49.16), -CH-OH (66.28), -CH-HN (68.45), py-C (125.18), py-C (141.52), py-C (149.12), -COOH (171.54).

The ^{13}C-NMR spectrum confirms the successful synthesis of *D*-Py-Thr free of impurity.

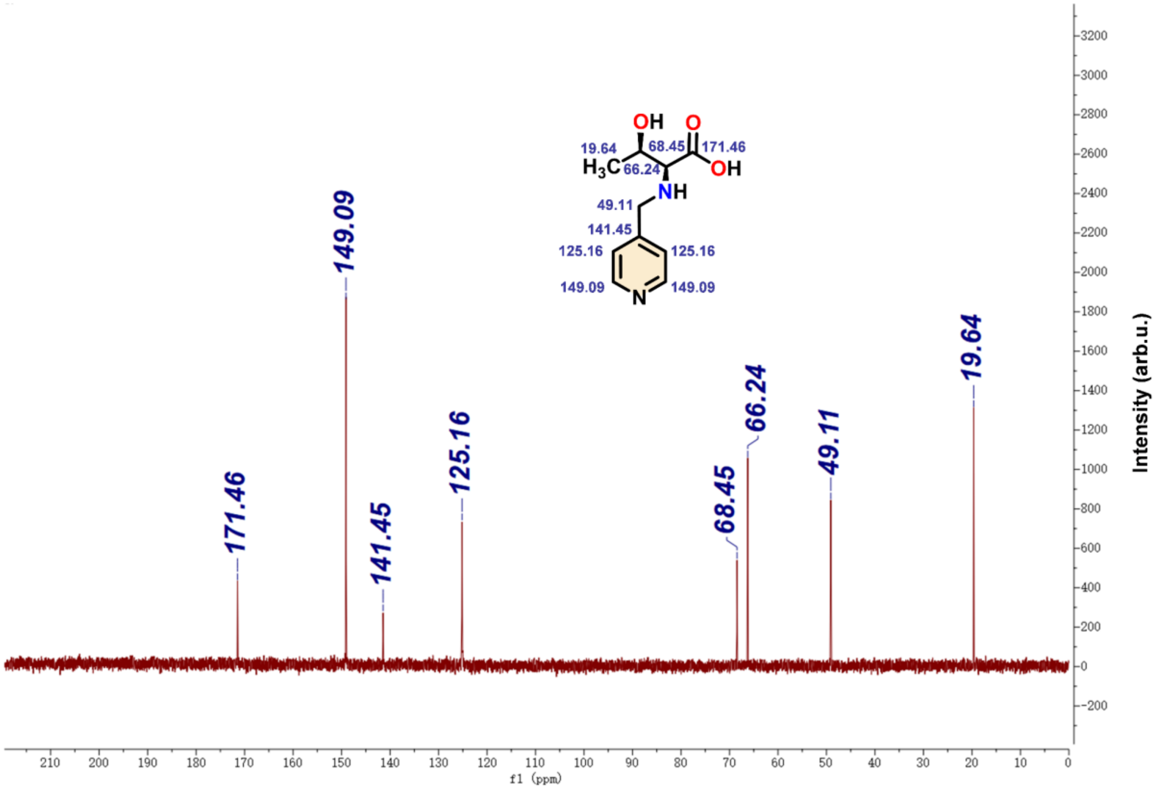

Supplementary Fig. 4. ^{13}C-NMR spectrum of *L*-Py-Thr. Peak assignments (ppm): -CH_3 (19.64), -CH_2 (49.11), -CH-OH (66.24), -CH-HN (68.45), py-C (125.16), py-C (141.45), py-C (149.09), -COOH (171.46).

The ^{13}C-NMR spectrum confirms the successful synthesis of *L*-Py-Thr free of impurity.

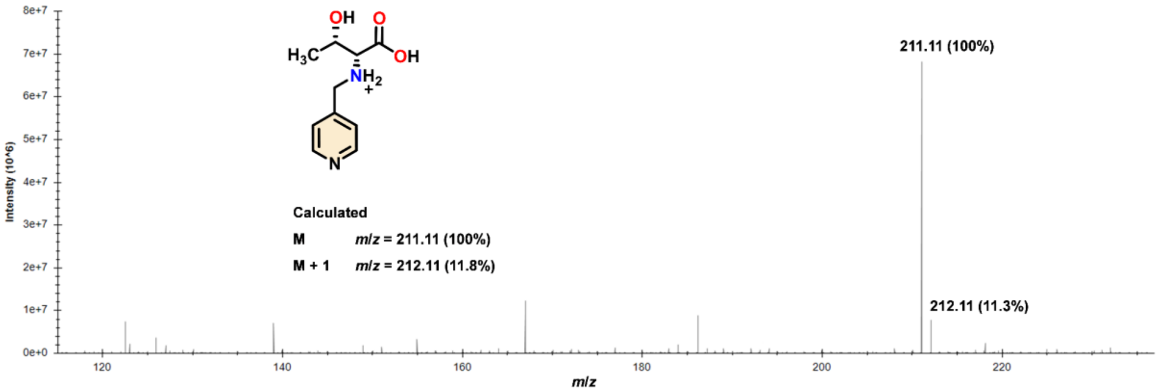

Supplementary Fig. 5. MS spectrum of *D*-Py-Thr. Obtained *m*/*z*: 211.11 (100%); *m*/*z*: 212.11 (11.3%). Calculated *m*/*z*: 211.11 (100%); *m*/*z*: 212.11 (11.8%).

As shown above, the peak of *m*/*z* = 211.11 is assigned to the molecular ion peak of protonated *D*-Py-Thr and the peak of *m*/*z* = 212.11 is assigned to the isotopic signal. The consistency between observed data with the simulated one further confirm the successful synthesis of *D*-Py-Thr ligand.

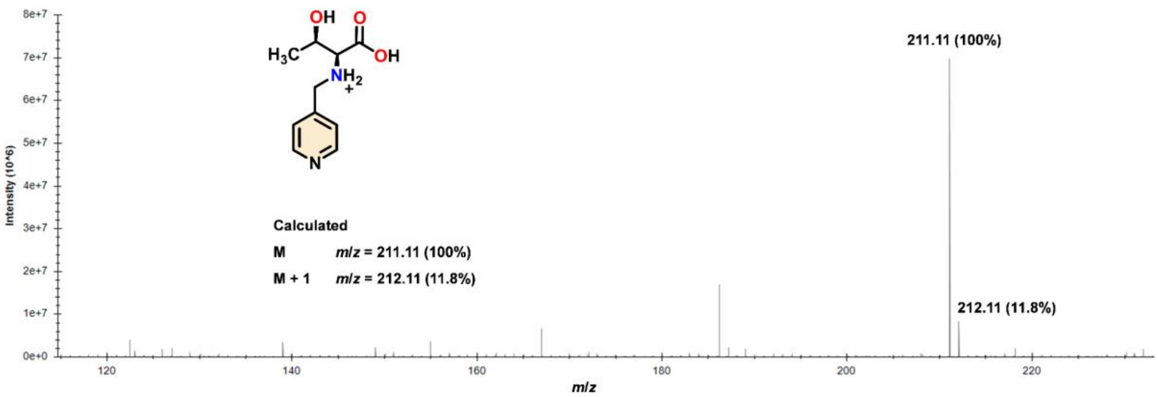

Supplementary Fig. 6. MS spectrum of *L*-Py-Thr. Obtained *m*/*z*: 211.11 (100%); *m*/*z*: 212.11 (11.8%). Calculated *m*/*z*: 211.11 (100%); *m*/*z*: 212.11 (11.8%).

Similarly, the peak of *m*/*z* = 211.11 is assigned to the molecular ion peak of protonated *L*-Py-Thr and the peak of *m*/*z* = 212.11 is assigned to the isotopic signal. The consistency between observed data with the simulated one further confirm the successful synthesis of *L*-Py-Thr ligand.

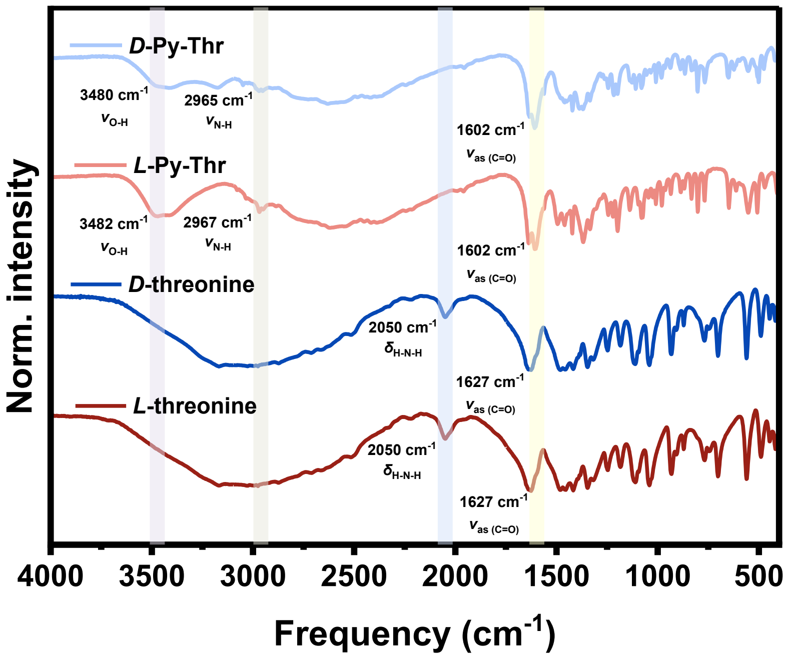

Supplementary Fig. 7. FT-IR spectra of *D*-Py-Thr (light blue curve), *L*-Py-Thr (light red curve), raw *D*-threonine (dark blue curve) and raw *L*- threonine (dark red curve). Source data are provided as a Source Data file.

The successful formation of *D*(*L*)-Py-Thr ligands is confirmed by the disappearance of the characteristic H-N-H bending vibration at 2050 cm^{-1} and the emergence of a new N-H stretching vibration at 2965 cm^{-1}. The asymmetric stretching vibration of the carboxyl group also undergoes a notable bathochromic shift from 1627 cm^{-1} in the starting *D*(*L*)-threonine to 1602 cm^{-1} in the derived *D*(*L*)-Py-Thr ligands.

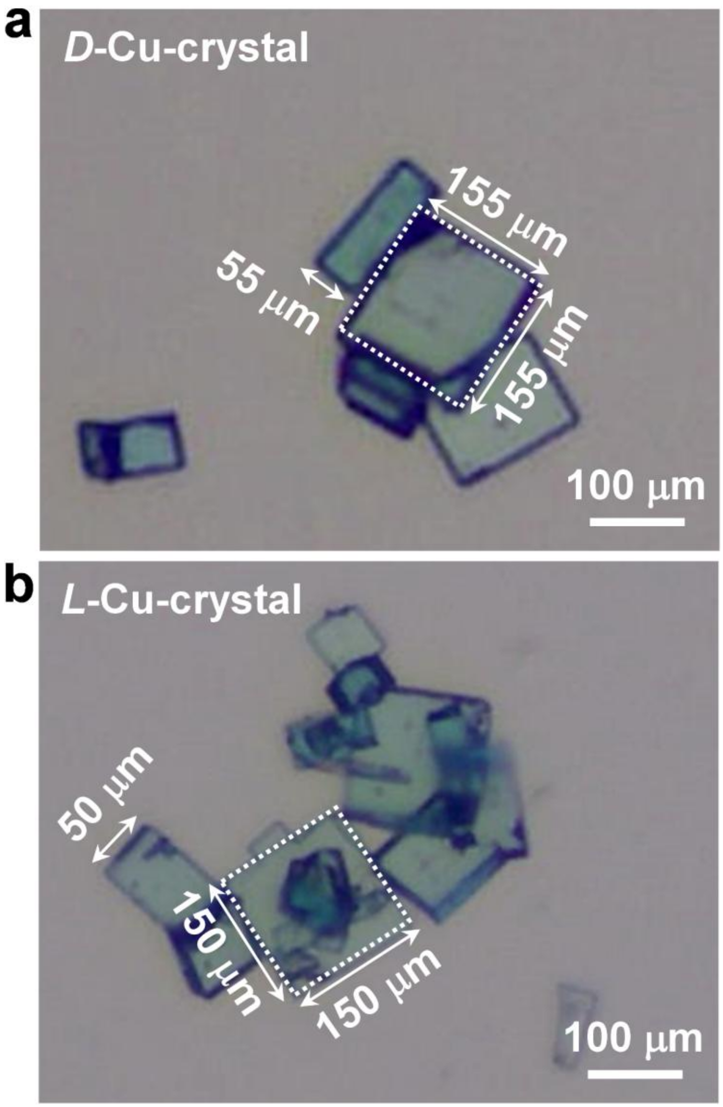

Supplementary Fig. 8. Morphological characterization of single-crystals. Optical microscopy images of (**a**) *D*-Cu-crystal and (**b**) *L*-Cu-crystal.

Rectangular blue crystals of *D*-Cu-crystal present a length and width of both 155 μm and a height of 55 μm. Similarly, rectangular blue crystals of *L*-Cu-crystal present a length and width of both 150 μm and a height of 50 μm.

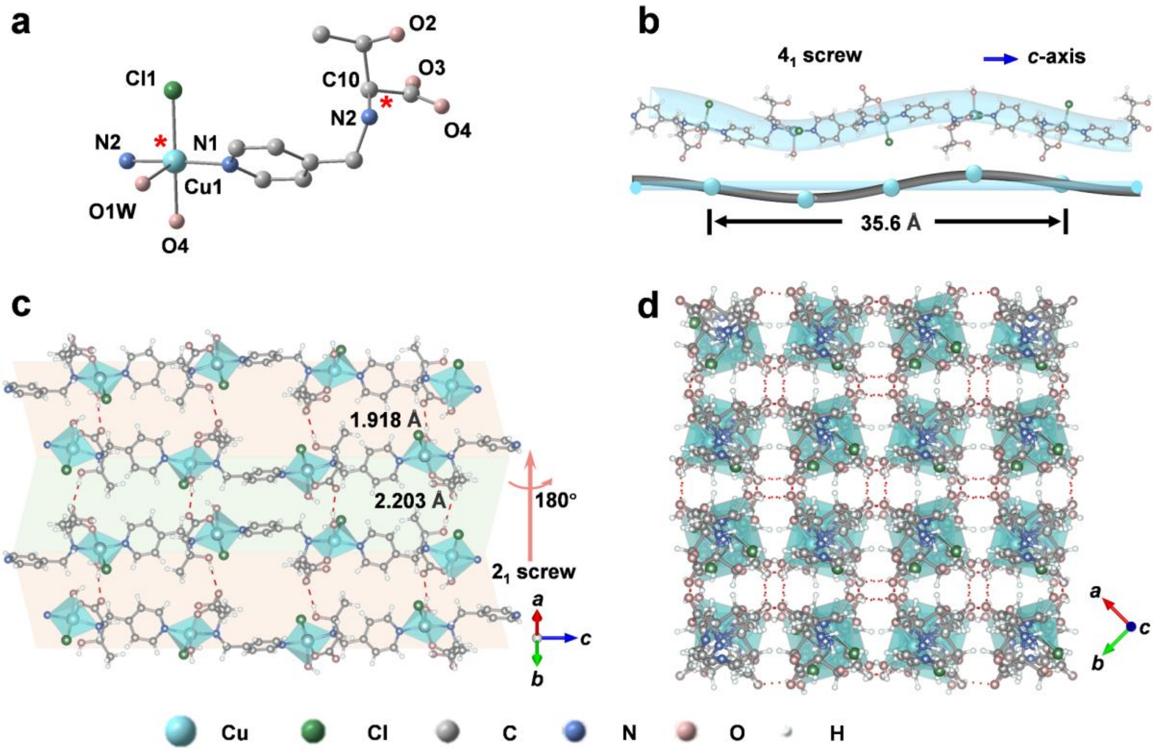

Supplementary Fig. 9. Dynamic evolution of assembly of *L*-Cu-crystal. a Asymmetric coordination mode of *L*-Cu-crystal with red asterisks indicating chiral centers. **b** The 1D helical unit featuring a left-handed 4_1 screw with a pitch of 35.6 Å along the *c*-axis direction. **c** 2D lamella structure assembled by interchain hydrogen bonding. **d** 3D framework structure projected in the *a*-*b* plane. Red dashed lines in (**c**) and (**d**) indicate hydrogen bonds. Element color: C is gray; O is pink; N is deep blue; H is white; Cl is green; Cu is light blue.

As illustrated in Supplementary Fig. 9a, *L*-Cu-crystal adopts an assembly mode identical to that of *D*-Cu-crystal (Fig. 2a) but with an inverted chiral configuration. Specifically, the Cu(II) center in *L*-Cu-crystal exhibits a five-coordinated asymmetric geometry, ligated by one pyridine nitrogen from *L*-Py-Thr, one amine nitrogen, one carboxylate oxygen from a second *L*-Py-Thr molecule, one chloride ion, and one water molecule. In contrast to the left-handed helix observed in *D*-Cu-crystal (Fig. 2b), the 1D spiral chain formed with *L*-Py-Thr adopts a right-handed 4_1 screw axis with a pitch of 35.6 Å along the *c*-axis (Supplementary Fig. 9b). These 1D helices further assemble into an ordered 2D sheet

structure stabilized by intermolecular hydrogen bonds between adjacent sheets (O_WH···O-C, 1.918 Å and O-H···O-C, 2.203 Å; Supplementary Fig. 9c). Owing to the tetragonal phase, these biomimetic hydrogen bonds are oriented in a perpendicular and intersecting manner within the crystallographic *a*-*b* plane, thereby constructing a three-dimensional (3D) hydrogen-bonded framework using the 1D [Cu(*L*-Py-Thr)(Cl)(H_2O)]$_\infty$ helical chain as the building unit (Supplementary Fig. 9d).

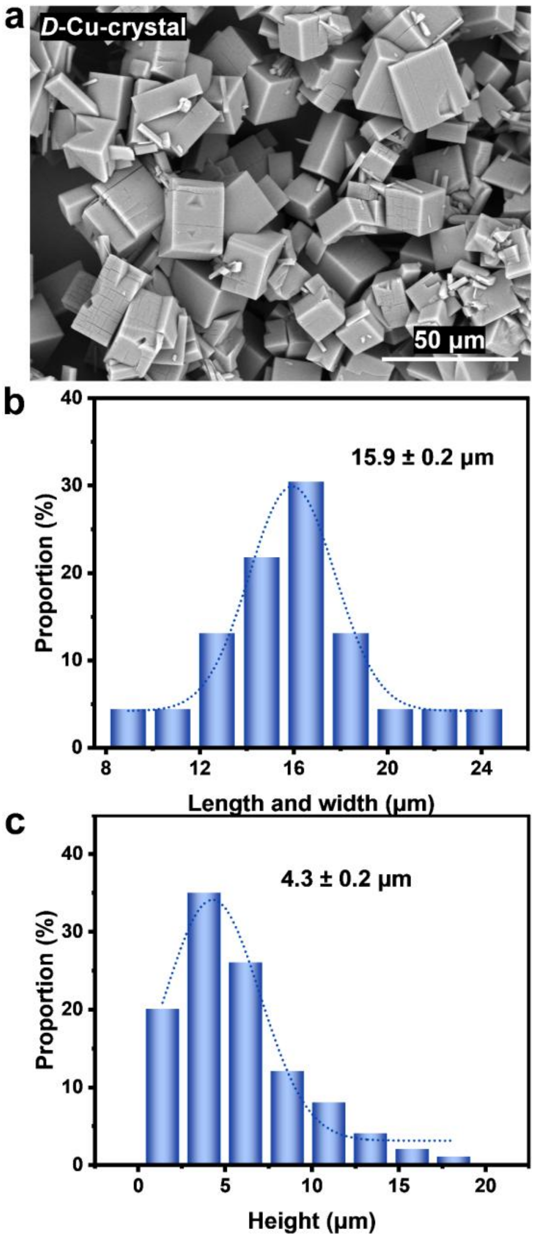

Supplementary Fig. 10. Morphological characterization of *D*-Cu-crystal. a SEM image of *D*-Cu-crystal. **b** Length and Width distribution of *D*-Cu-crystal. **c** Height distribution of *D*-Cu-crystal. (n = 20 crystals) Source data are provided as a Source Data file.

According to statistical counting, *D*-Cu-crystal powder sample presents an average length and width of 15.9 μm and an average height of 4.3 μm.

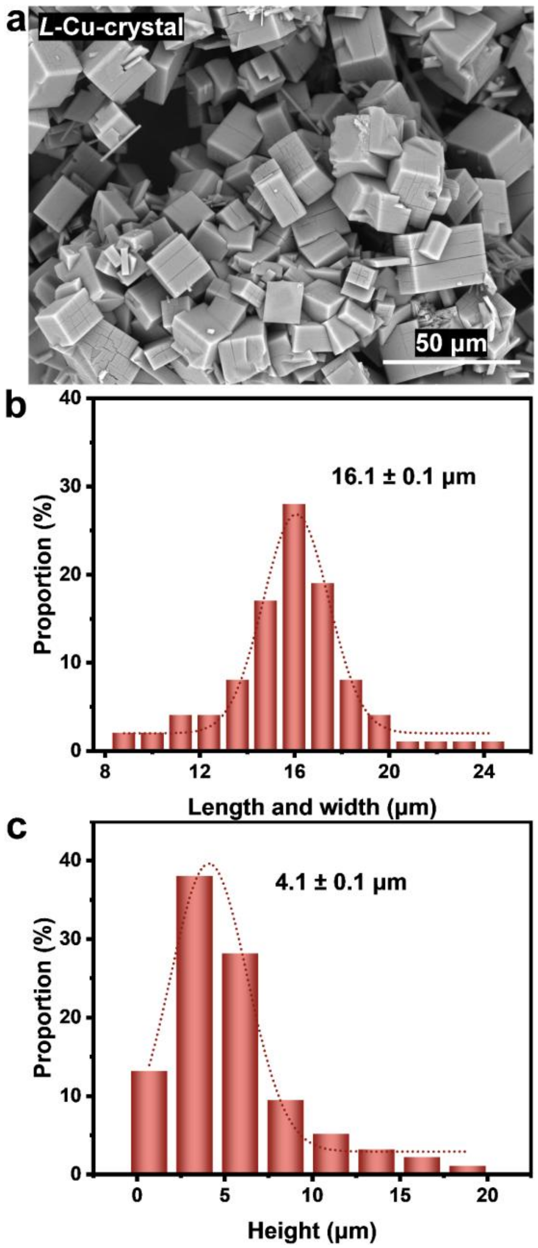

Supplementary Fig. 11. Morphological characterization of *L*-Cu-crystal. a SEM image of *L*-Cu-crystal. **b** Length and Width distribution of *L*-Cu-crystal. **c** Height distribution of *L*-Cu-crystal. (n = 20 crystals) Source data are provided as a Source Data file.

According to statistical counting, *L*-Cu-crystal powder sample presents an average length and width of 16.1 μm and an average height of 4.1 μm.

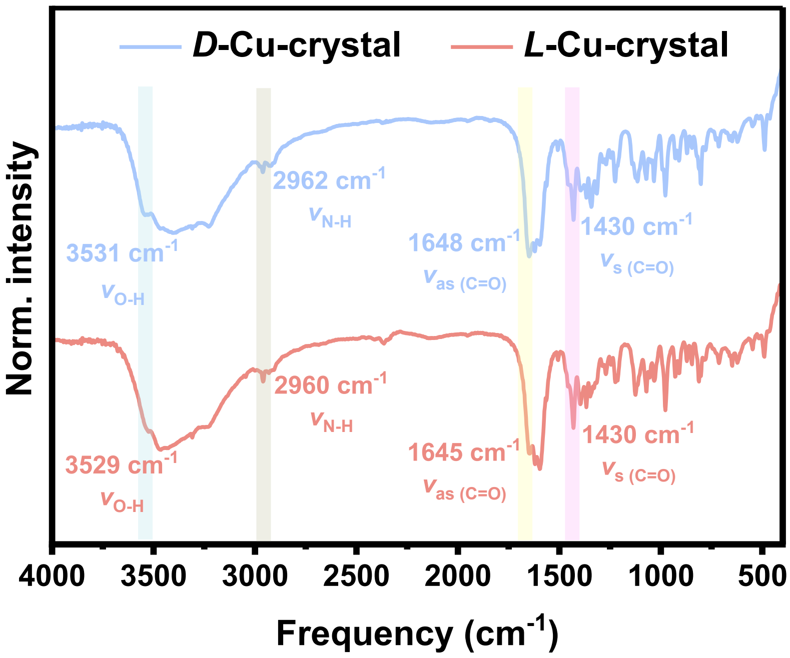

Supplementary Fig. 12. FT-IR spectra of *D*-Cu-crystal (blue curve) and *L*-Cu-crystal (red curve). Source data are provided as a Source Data file.

The as-synthesized *D*-Cu-crystal and *L*-Cu-crystal exhibit nearly identical FT-IR absorption spectra. Using *D*-Cu-crystal as a representative example, the absorption peak observed at 3531 cm^{-1} is assigned to the stretching vibration of -OH group, while the peak at 2962 cm^{-1} corresponds to the stretching vibration of -NH group. Additionally, the peaks centered at 1648 cm^{-1} and 1430 cm^{-1} are attributed to the asymmetric and symmetric stretching vibrations of the carboxyl group, respectively.

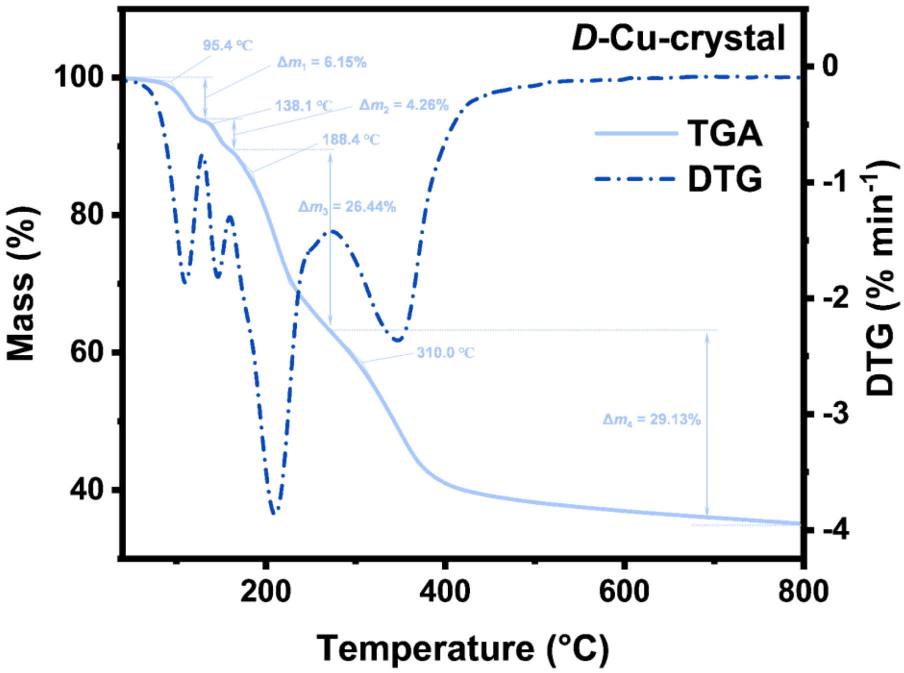

Supplementary Fig. 13. TGA curve (blue solid line) and DTG curve (blue dashed line) of *D*-Cu-crystal. Source data are provided as a Source Data file.

On basis of the above result, *D*-Cu-crystal begin to lose residual solvent at 95.4 ºC and coordinated water molecules at 138.1 ºC, respectively. As the temperature rises to 188.4 ºC, the framework begins to gradually decompose by losing organic ligands.

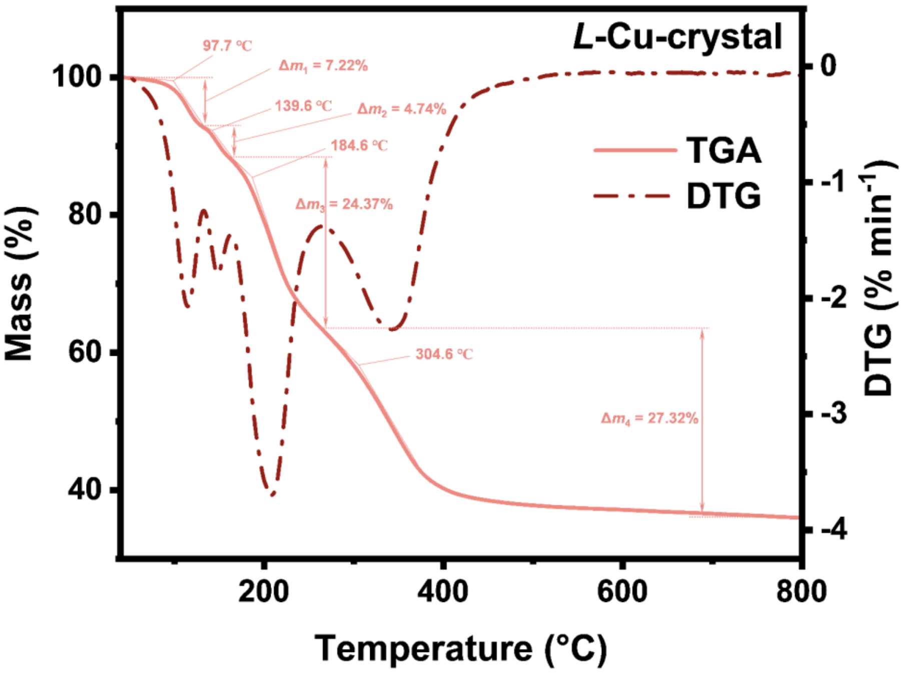

Supplementary Fig. 14. TGA curve (red solid line) and DTG curve (red dashed line) of ***L***-Cu-crystal. Source data are provided as a Source Data file.

On basis of the above result, *L*-Cu-crystal begin to lose the residual solvent at 97.7 ºC and coordinated water molecules at 139.6 ºC, respectively. As the temperature rises to 184.6 ºC, the framework begins to gradually decompose by losing organic ligands.

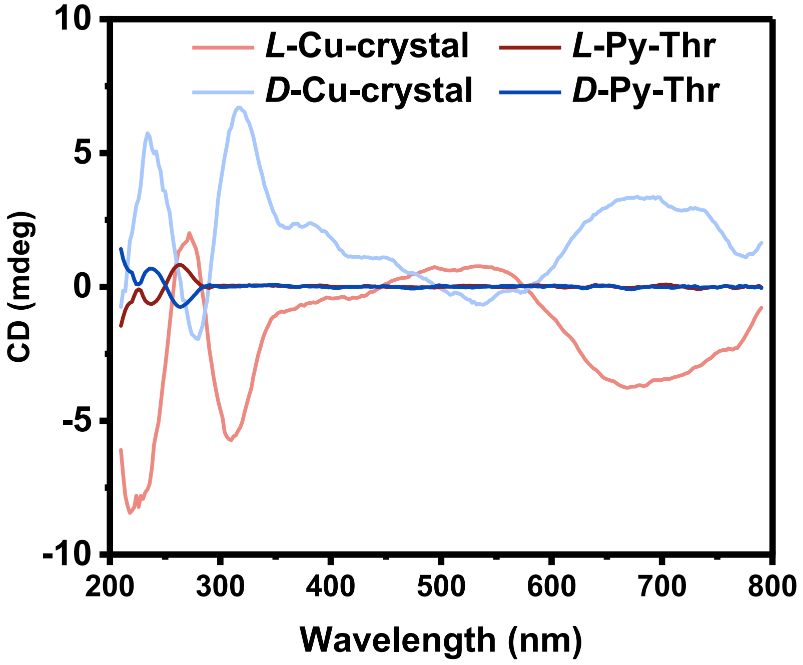

Supplementary Fig. 15. CD spectra of *D*(*L*)-Cu-crystals and *D*(*L*)-Py-Thr ligands at the same concentration. Source data are provided as a Source Data file.

In specification, the CD peak centered at 220 nm is attributed to the $n \rightarrow \pi^*$ transition of the carboxylate group[1]. While CD signals around 270 nm are attributed to the $\pi \rightarrow \pi^*$ and $n \rightarrow \pi^*$ electron transitions of the pyridyl ring which are involved in a homohelical configuration[1]. In sharp contrast, weak chiroptical responses around 260 nm are observed for raw *D*(*L*)-Py-Thr ligands at the optical window of pyridyl groups due to lacking such helical configuration. On account of the asymmetric coordination mode of central Cu(II), CD signals near 300 nm arise from the ligand-to-metal charge transfer (LMCT) process[2] while CD responses appearing in visible regions arise from *d*-*d* transitions of asymmetric Cu(II) center featuring an incompletely occupied $3d^9$ configuration[3].

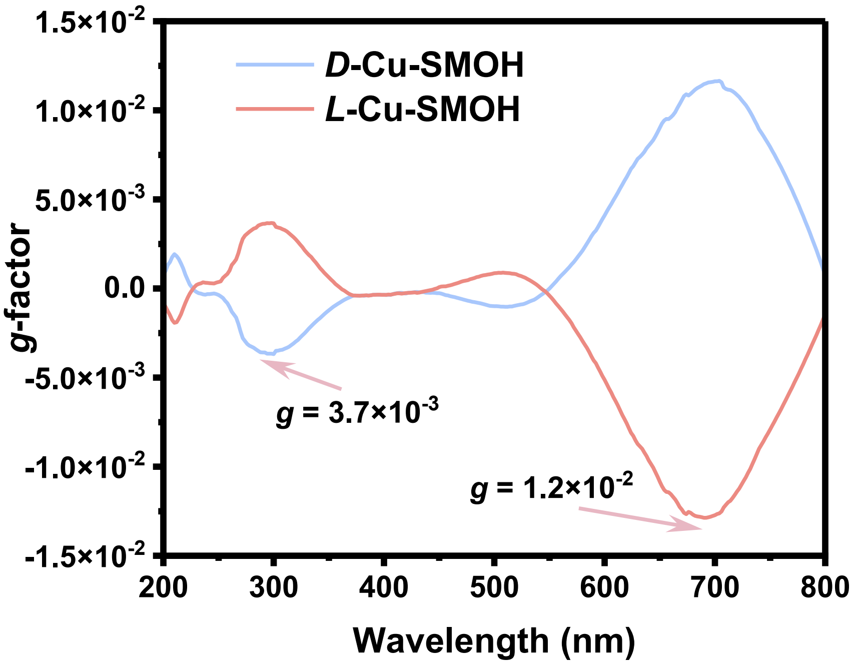

Supplementary Fig. 16. The asymmetric *g*-factor of *D*(*L*)-Cu-SMOHs. Source data are provided as a Source Data file.

The corresponding asymmetric *g*-factors ($g_{\mathrm{Abs}} = \frac{\mathrm{CD}}{32980\times\mathrm{Abs}}$) of *D*(*L*)-Cu-SMOHs are calculated up to be 3.7×10^{-3} and 1.2×10^{-2} centered at 302 nm and 694 nm, respectively.

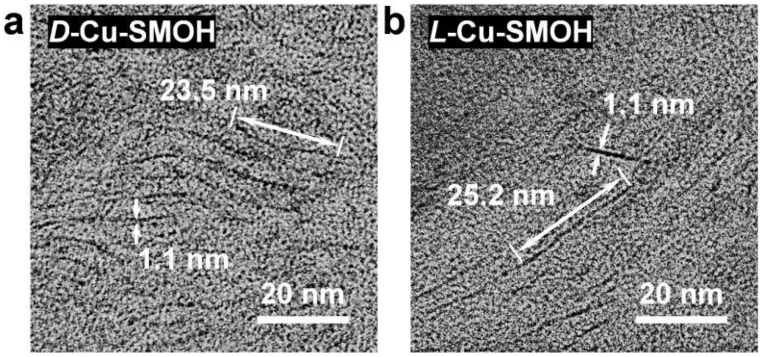

Supplementary Fig. 17. HR-TEM image of *D*(*L*)-Cu-SMOHs under physiological conditions (10 mM physphosphate-buffer, PB, pH=7.4). a *D*-Cu-SMOH. **b** *L*-Cu-SMOH. Note that minor noise spots may originate from residual buffer salts.

HR-TEM analysis of *D*-Cu-SMOH and *L*-Cu-SMOH after incubation in PB at 37 °C for a period representative of the biological assays confirmed that the single-unit helical morphologies with averaged diameters of 1.1 nm and averaged lengths of 23.5 nm and 25.2 nm, respectively, was well-maintained that were not significantly different from those in pure water.

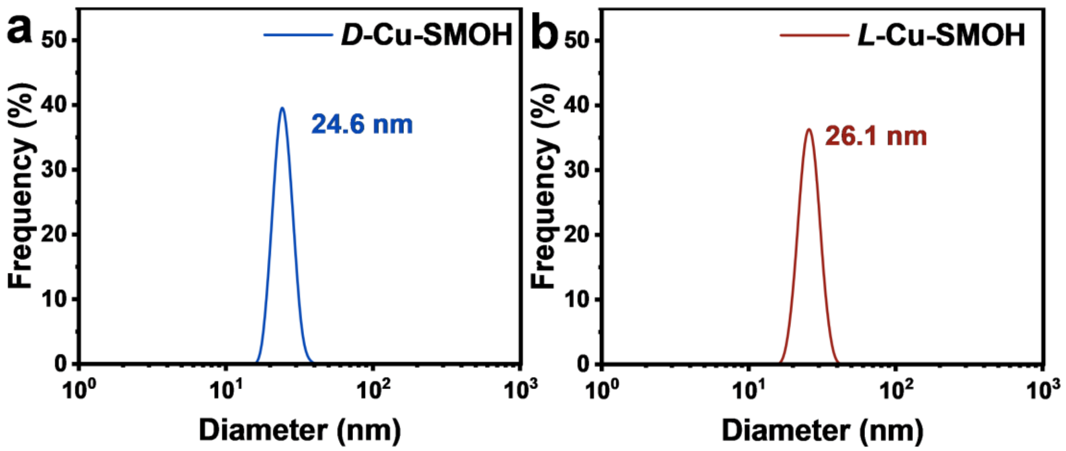

Supplementary Fig. 18. DLS characterization of *D*(*L*)-Cu-SMOHs under physiological conditions (10 mM physphosphate-buffer, PB, pH=7.4). a *D*-Cu-SMOH. **b** *L*-Cu-SMOH. Source data are provided as a Source Data file.

DLS measurements performed in PB showed that the *D*-Cu-SMOH (24.6 nm) and *L*-Cu-SMOH (26.1 nm) remained as well-dispersed nano-helixes with hydrodynamic diameters that were not significantly different from those measured in pure water.

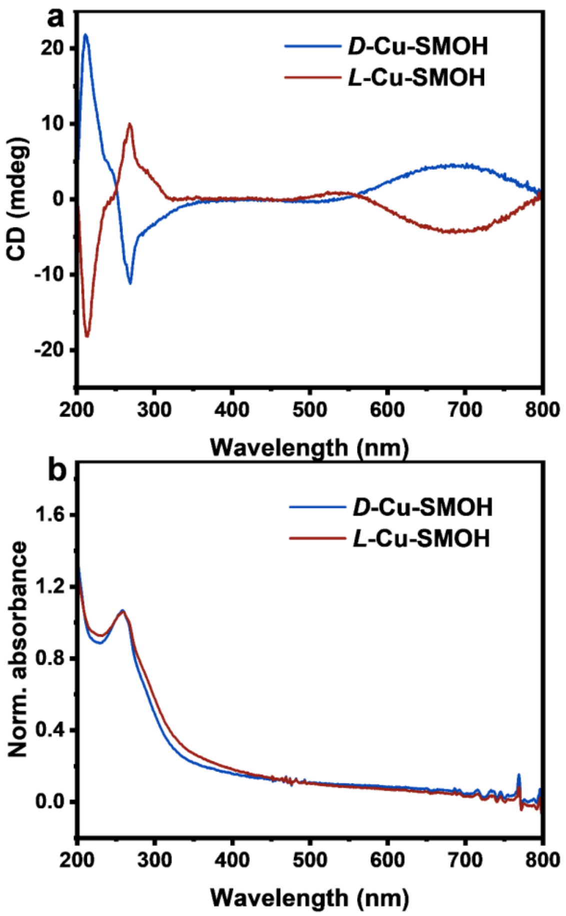

Supplementary Fig. 19. Optical activity of *D*(*L*)-Cu-SMOHs under physiological conditions (10 mM physphosphate-buffer, PB, pH=7.4). a CD spectra. **b** Corresponding UV-vis absorbance spectra. Source data are provided as a Source Data file.

The CD spectra of *D*-Cu-SMOH and *L*-Cu-SMOH recorded in PB were found to be remarkably similar to those obtained in pure water. The characteristic Cotton effects, indicative of their chiral helical structures, were well-preserved with no significant changes in signal position or intensity.

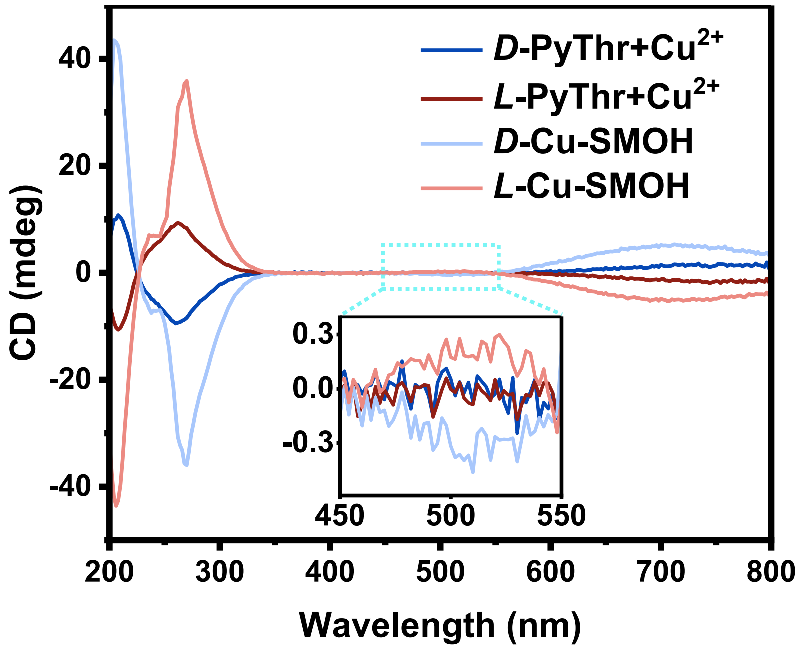

196

197 **Supplementary Fig. 20.** CD spectra of *D*(*L*)-Py-Thr-Cu^{2+} complex as well as *D*(*L*)-Cu-
198 SMOHs at the same concentration. Source data are provided as a Source Data file.

199

200 As shown in Supplementary Fig. 20, *D*(*L*)-Cu-SMOHs exhibited distinctly different
201 chiroptical responses compared to the simple *D*(*L*)-Py-Thr-Cu(II) complex within the 200-
202 800 nm absorption range at identical concentrations. Additionally, the inset shows CD
203 responses in the visible region (450-550 nm). Clearly, *D*(*L*)-Cu-SMOHs demonstrated
204 mirror-image CD signals whereas no such signals were observed for the *D*(*L*)-Py-Thr-Cu(II)
205 complex.

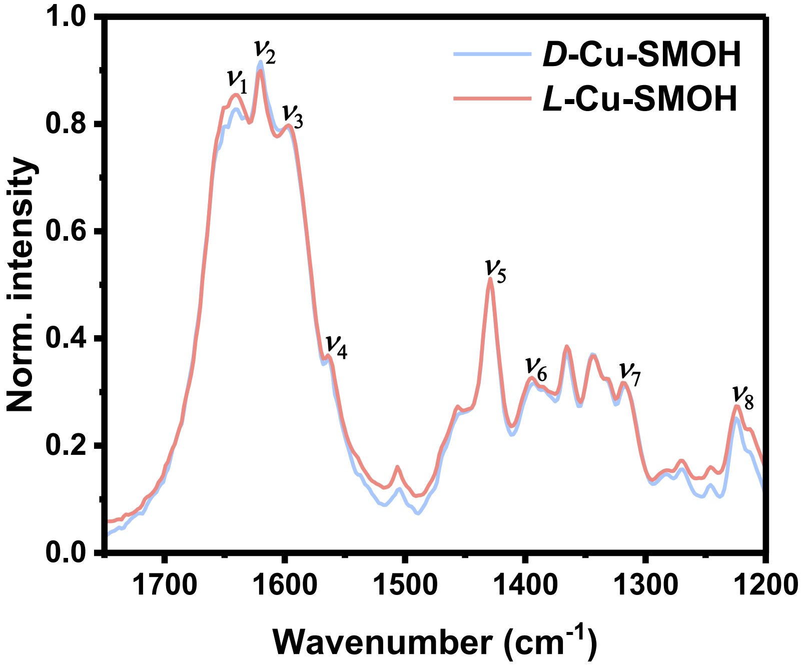

Supplementary Fig. 21. IR absorbance spectra of *D*(*L*)-Cu-SMOHs. Source data are provided as a Source Data file.

The enantiomeric *D*-Cu-SMOH and *L*-Cu-SMOH exhibit pronounced split-type Cotton effects (purple shaded region, Fig 2m) at 1641 (v_1), 1430 (v_5), and 1317 (v_7) cm^{-1}. These signals are attributed to the C=O and C−O stretching vibrations originating from the carboxylate (COO^-) group[4,5] (Supplementary Table 14). Additionally, characteristic positive or negative VCD signals (blue shaded region, Fig 2m) are observed, arising from the aromatic pyridine C=C/C=N bond vibrations[5,6] (v_2, v_3, v_4, and v_6) and the C-H vibrations of the pyridine ring[6,7] (v_8), which were virtually mirror images of each other.

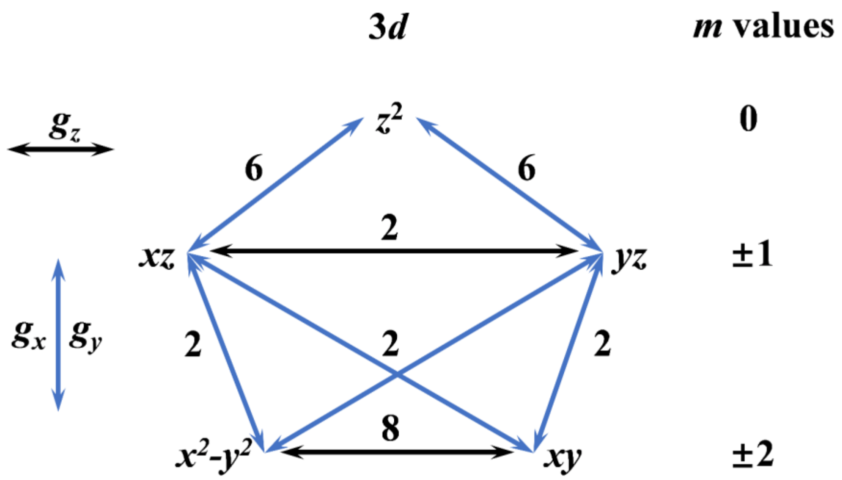

Supplementary Fig. 22. The energy level splitting of the 3*d* orbitals in the crystal field. The transverse coupling (black arrows) between orbitals corresponds to "g_z", longitudinal and oblique couplings (blue arrows) correspond to "g_x" or "g_y".

Among the 75 integrals $\langle n|\hat{\mathrm{L}}_i|0\rangle$ presented in Supplementary Table 15, only 16 integrals resulted in non-zero values, specifically $\pm\mathrm{i}$, $\pm\sqrt{3}\mathrm{i}$, or $\pm 2\mathrm{i}$. Consequently, "$2\langle 0|\hat{\mathrm{L}}_i|n\rangle\langle n|\hat{\mathrm{L}}_i|0\rangle$" is restricted to the value of 2, 6, or 8. These numerical values correspond precisely to the numbers assigned to each arrow in the above magic pentagon. Notably, when d_{z^2} is the ground state, $\hat{\mathrm{L}}_x|d_{z^2}\rangle$ (i.e., g_x) and $\hat{\mathrm{L}}_y|d_{z^2}\rangle$ (i.e., g_y) have operators of $-\sqrt{3}\mathrm{i}$ and $\sqrt{3}\mathrm{i}$, which couples to d_{yz} and d_{xz}, respectively, both corresponding to the blue arrows with value of 6. Whereas the operator of $\hat{\mathrm{L}}_z|d_{z^2}\rangle$ (i.e., g_z) is 0 and thus resulting $g_z \approx g_e$.

When $d_{x^2-y^2}$ is the ground state, both $\hat{\mathrm{L}}_x|d_{x^2-y^2}\rangle$ (i.e. g_x) and $\hat{\mathrm{L}}_y|d_{x^2-y^2}\rangle$ (i.e., g_y) have the operator of $-\mathrm{i}$, which couples to d_{yz} and d_{xz}, respectively, both corresponding to blue arrows with value of 2. While $\hat{\mathrm{L}}_z|d_{x^2-y^2}\rangle$ (i.e., g_z) has the operator of $2\mathrm{i}$, which couples to d_{xy} and corresponds to the black arrow with value of 8.

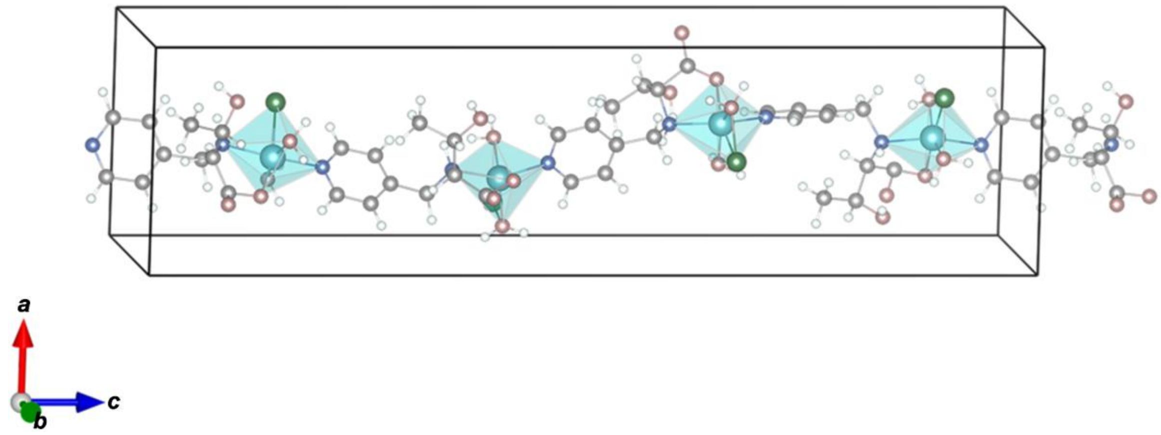

Supplementary Fig. 23. Structural modeling of *D*-Cu-SMOH-H_2O.

As shown in Supplementary Fig. 23, the Cu(II) center in the optimized *D*-Cu-SMOH model transform from the original triangular bipyramidal (TBP) fashion to an elongated octahedral (EO) fashion after coordination of additional water molecule.

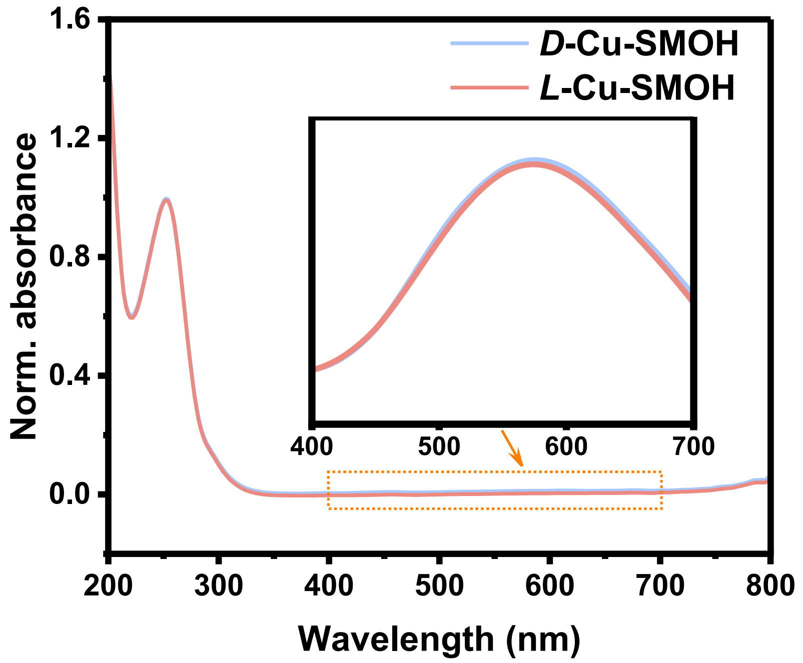

Supplementary Fig. 24. UV-vis absorbance spectra of *D*-Cu-SMOH and *L*-Cu-SMOH. Source data are provided as a Source Data file.

The spectra exhibit intense absorption bands below 400 nm attributed to LMCT and intra-ligand ($\pi \rightarrow \pi^*$ and $n \rightarrow \pi^*$) transitions[1,2]. Crucially, a broad, lower-intensity absorption band is observed in the visible region. This feature is characteristic of the *d*-*d* electronic transitions of the Cu(II) center in its coordination environment[3]. The presence of these *d*-*d* transitions in this region is consistent with our EPR spectroscopic data (Fig. 3d), which also indicates an EO Cu(II) geometry.

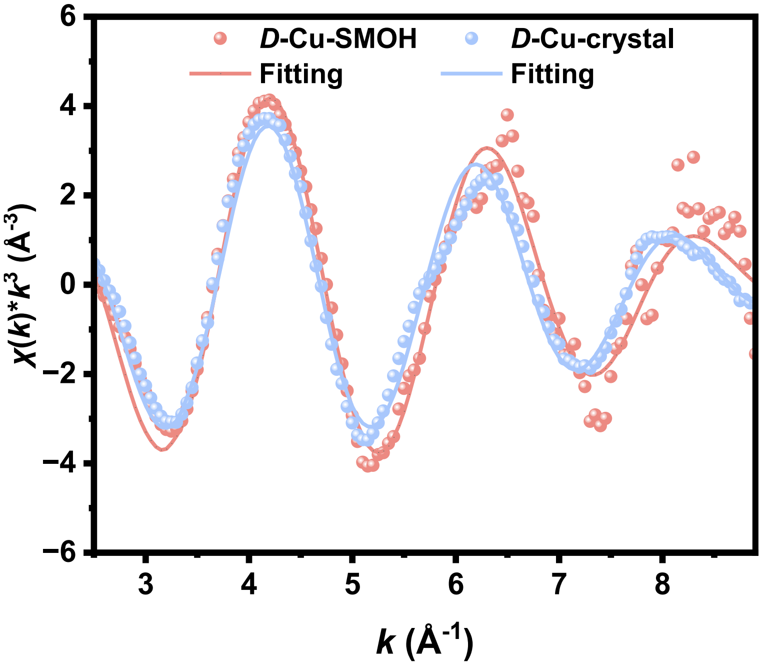

Supplementary Fig. 25. The *k*-space oscillations spectra from EXAFS of *D*-Cu-crystal and *D*-Cu-SMOH. Source data are provided as a Source Data file.

As shown in Supplementary Fig. 25, the oscillation modes of *D*-Cu-SMOH and *D*-Cu-crystal in the *k*-space are almost the same with minor intensity differences.

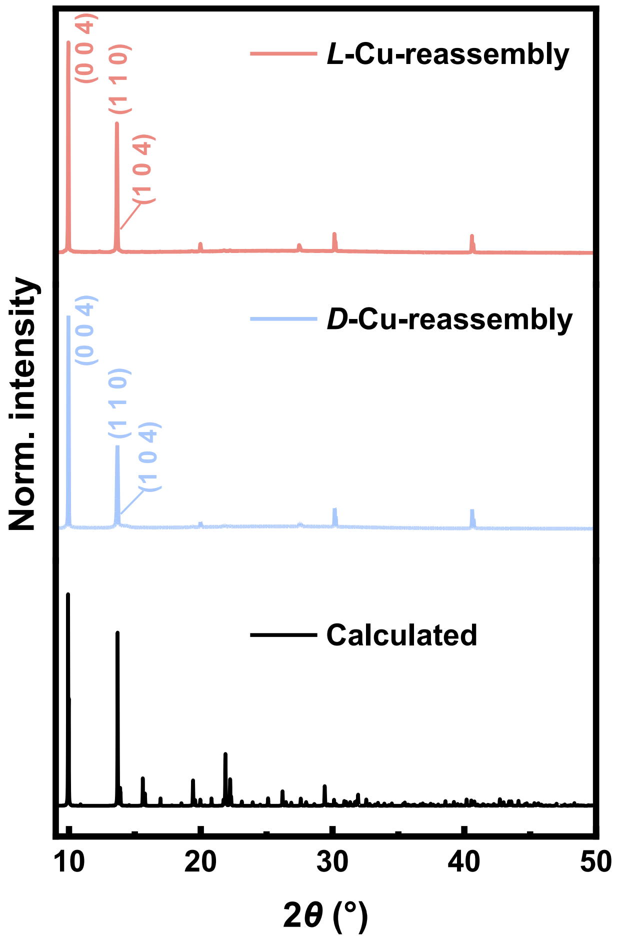

Supplementary Fig. 26. PXRD patterns of *D*(*L*)-Cu-reassemblies alongside calculated patterns. Source data are provided as a Source Data file.

This reassembly was unequivocally confirmed by PXRD analysis. The PXRD pattern of the reassembled material, obtained after the addition of ACN to a final ACN/H_2O ratio of 1:1 v/v and subsequent centrifugation closely matched both the pattern calculated from the SXRD data of the original crystalline *D*(*L*)-Cu-SMOHs.

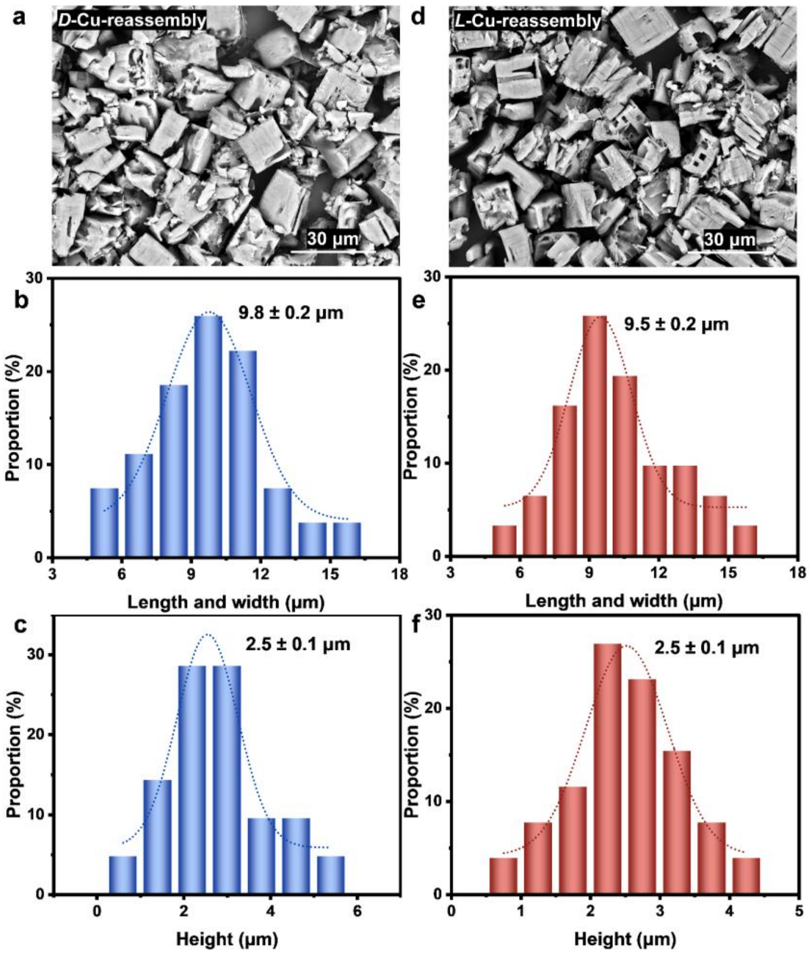

Supplementary Fig. 27. Morphological characterization of *D*(*L*)-Cu-reassemblies. a SEM image of *D*-Cu-reassembly. **b** Length and width distribution of *D*-Cu-reassembly. **c** Height distribution of *D*-Cu-reassembly. **d** SEM image of *L*-Cu-reassembly. **e** Length and width distribution of *L*-Cu-reassembly. **f** Height distribution of *L*-Cu-reassembly. (n = 20 reassembly crystals) Source data are provided as a Source Data file.

The SEM images show that the reassembled crystals are identical to the original bulk crystals, an observation that further confirms the successful reassembly of the 3D frameworks. According to statistical counting, *D*-Cu-reassembly sample presents an average length and width of 9.8 μm and an average height of 2.5 μm; while *L*-Cu-reassembly sample presents an average length and width of 9.5 μm and an average height of 2.5 μm.

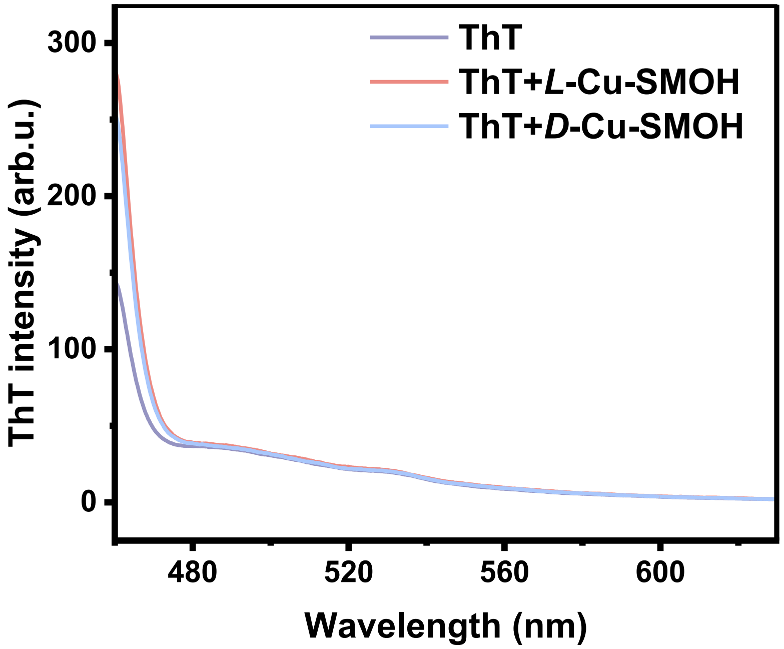

Supplementary Fig. 28. ThT fluorescence spectroscopy of raw ThT and ones in the presence of only D-Cu-SMOH or L-Cu-SMOH. Source data are provided as a Source Data file.

It is evident that *D*-Cu-SMOH and *L*-Cu-SMOH themselves both show neglectable FL influence on ThT experiments.

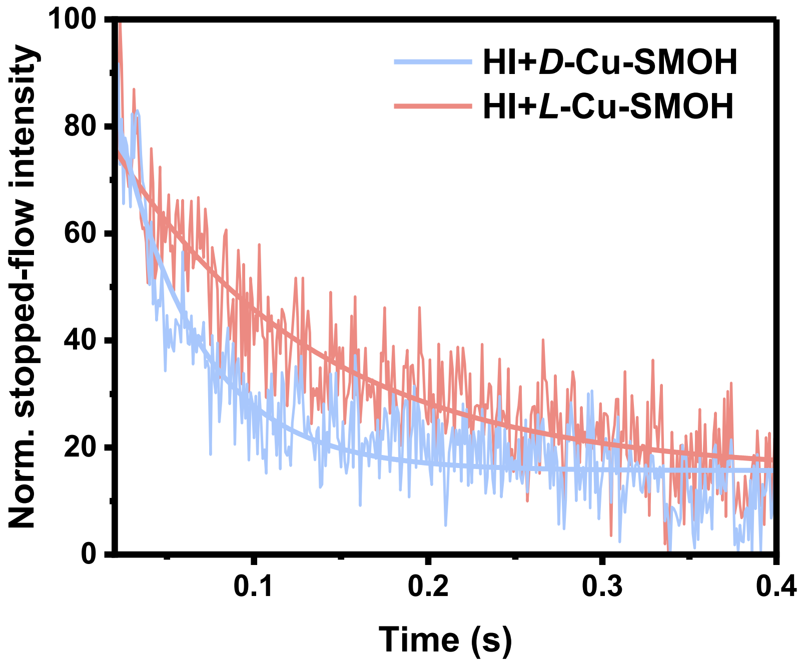

Supplementary Fig. 29. Stopped-flow fluorescence spectroscopy of HI in the presence of *D*-Cu-SMOH or *L*-Cu-SMOH. Source data are provided as a Source Data file.

The fluorescence decay kinetics of *D*-Cu-SMOH exhibit a significantly faster profile compared to its *L*-Cu-SMOH enantiomer. The kinetics were analyzed by fitting to a single-exponential function, as described in Supplementary Eq. (1)[8]:

$$F = c + A\exp(-kt) \tag{1}$$

Where F represents the change in HI fluorescence intensity; c is the fitting intercept, corresponding to the baseline offset of the kinetic curve; A and k denote the amplitude and observed rate constant, respectively; and t is the reaction time.

According to the fitting results, *D*-Cu-SMOH (21.70 s^{-1}) represents a faster binding rate compared to *L*-Cu-SMOH (8.56 s^{-1}).

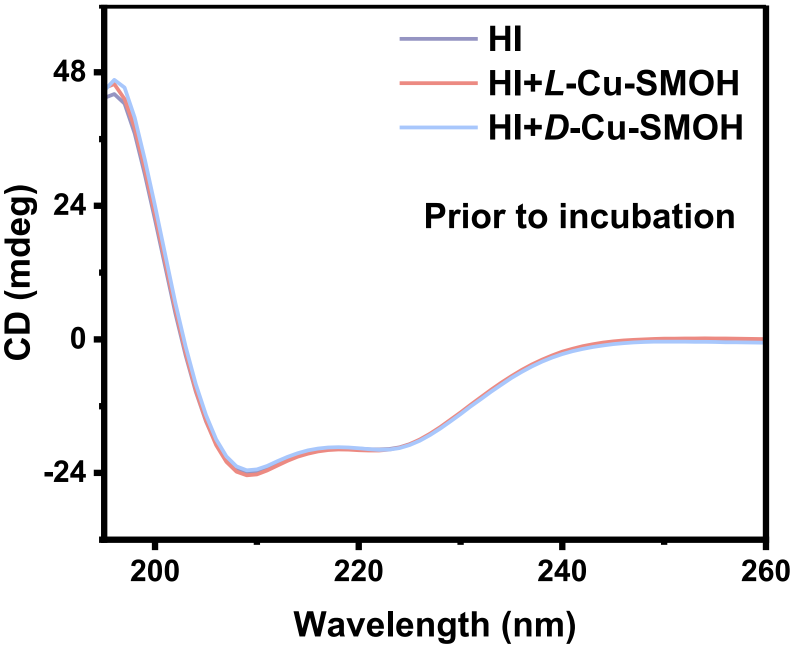

Supplementary Fig. 30. Control CD spectra of HI in the presence/absence of *D*-Cu-SMOH or *L*-Cu-SMOH, recorded immediately after mixing and prior to incubation. These data confirm the absence of immediate inhibitor-induced changes to HI's secondary structure. Source data are provided as a Source Data file.

Prior to incubation, the native HI monomer exhibits a negative doublet in the CD spectrum with minima at approximately 208 and 222 nm, which are diagnostic signatures of α-helical secondary structures. The *D*(*L*)-Cu-SMOHs additives demonstrate negligible CD interference under these experimental conditions, owing to the low working concentration employed in this study.

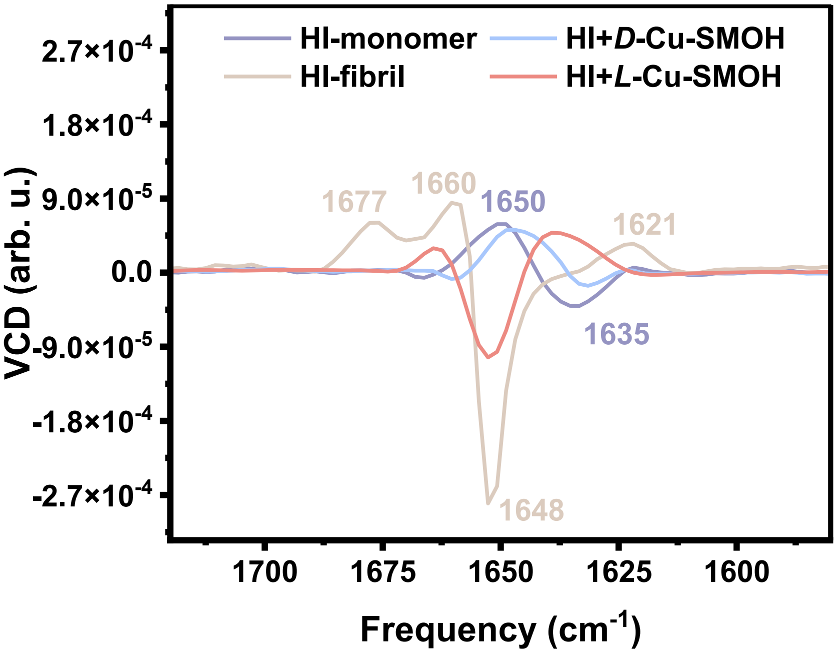

Supplementary Fig. 31. VCD spectra of HI without/with *D*(*L*)-Cu-SMOHs incubation. Source data are provided as a Source Data file.

The VCD spectra clearly show that native HI monomer exhibits α-helical characteristics (VCD bands at 1650(+) and 1635(-) cm^{-1}), which transform into distinct β-sheet signatures (VCD bands at 1677(+), 1660(+), 1648(-), and 1621(+) cm^{-1}) upon fibrillization[9-13]. Crucially, in the presence of *D*-Cu-SMOH, HI largely retains its native-like VCD profile, indicating potent inhibition of β-sheet formation. In contrast, *L*-Cu-SMOH allows partial conversion to β-sheet structures, as evidenced by intermediate VCD features (peak red shifts and emergence of weak β-sheet signals). These VCD results provide direct molecular-level evidence for the superior and enantioselective efficacy of *D*-Cu-SMOH in preserving the native conformation of HI, strongly corroborating our CD secondary structure findings.

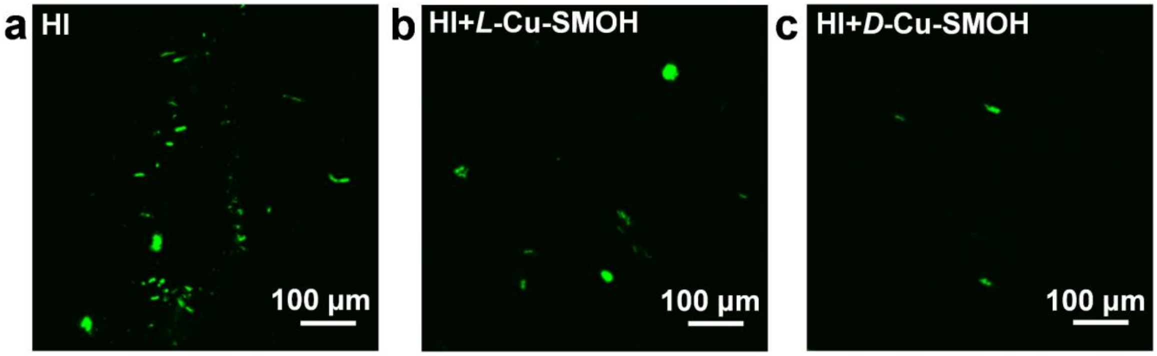

Supplementary Fig. 32. ThT fluorescence CLSM images. Representative images show amyloid aggregates of HI alone (**a**) and *L*-Cu-SMOH (**b**) or *D*-Cu-SMOH (**c**). Scale bar is 100 μm.

As evidenced by Supplementary Fig. 32a, substantial HI aggregates demonstrating intense fluorescence emission emerge after incubation. Comparative analysis reveals a marked reduction in aggregates in the sample incubated with for *D*(*L*)-Cu-SMOHs (Supplementary Fig. 32b, c). More strikingly, HI aggregates are virtually undetectable in the *D*-Cu-SMOH treated HI sample.

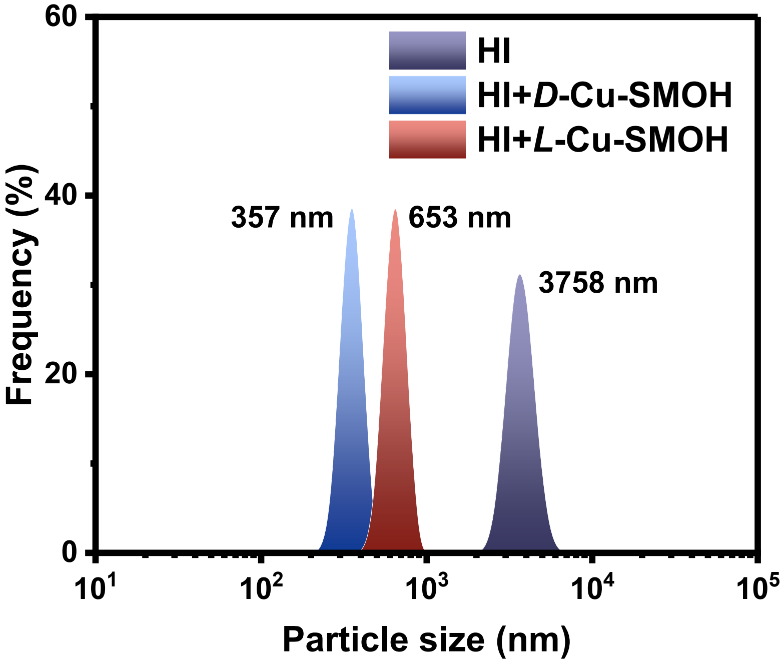

Supplementary Fig. 33. Size distribution of HI incubated in the presence/absence of *D*-Cu-SMOH or *L*-Cu-SMOH. Source data are provided as a Source Data file.

Mature amyloid fibrils formed in the absence of *D*-Cu-SMOH or *L*-Cu-SMOH exhibit a mean hydrodynamic radius of 3758 nm. Strikingly, *D*-Cu-SMOH and *L*-Cu-SMOH co-incubated HI sample demonstrate dramatically reduced hydrodynamic sizes of 357 nm and 653 nm, respectively.

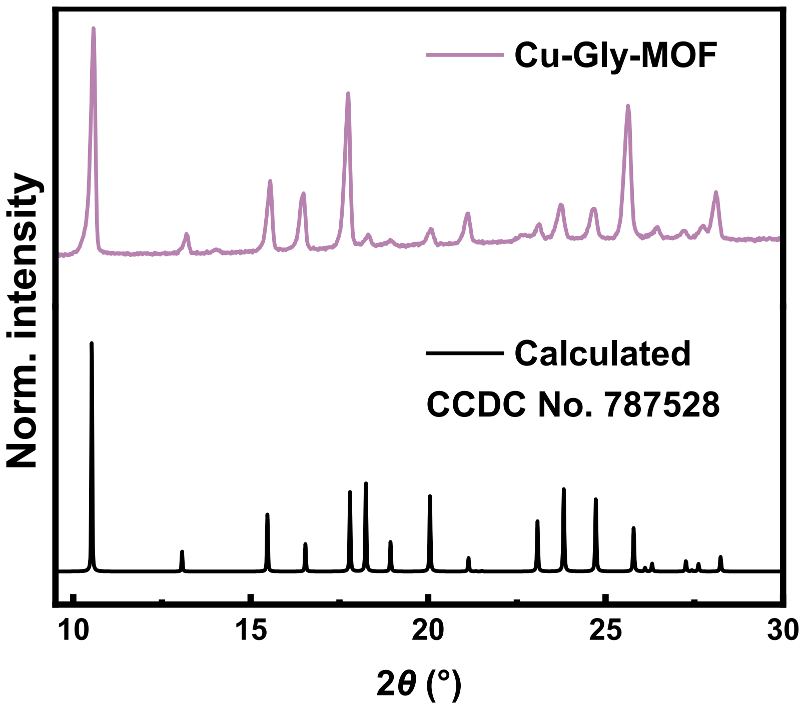

Supplementary Fig. 34. PXRD patterns of Cu-Gly-MOF (CCDC 787528) alongside calculated patterns. Source data are provided as a Source Data file.

The Cu-Gly-MOF (CCDC 787528) was synthesized according to a literature procedure[14,15], and its successful synthesis and phase purity were confirmed by comparing its PXRD pattern with the pattern calculated from its reported SXRD data.

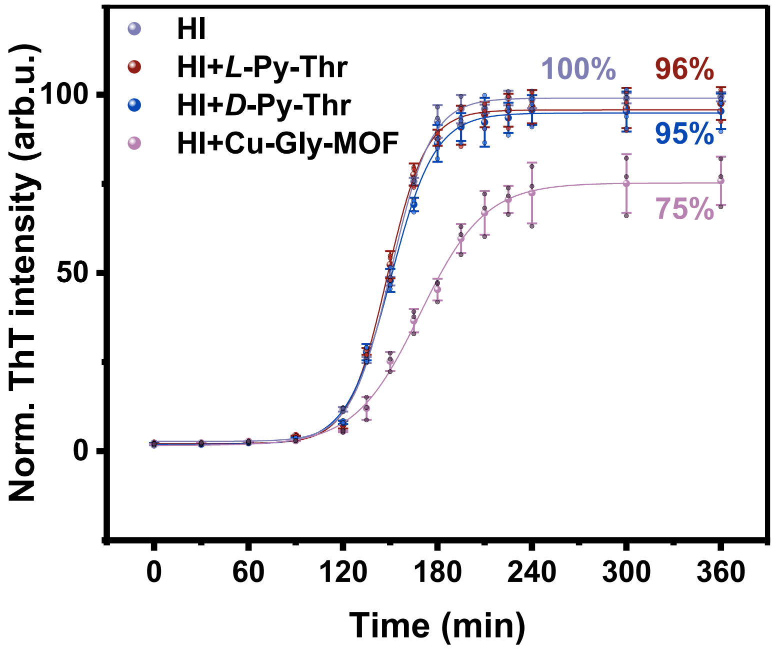

Supplementary Fig. 35. Kinetics of HI fibrillization monitored by the ThT assay in the absence/presence of *D*(*L*)-Py-Thr ligands or Cu-Gly-MOF. Error bars indicate the s.d. (n = 3 independent samples). Source data are provided as a Source Data file.

The ThT fluorescence intensity in the presence of *D*-Py-Thr or *L*-Py-Thr was nearly identical to that of the HI-only control group, indicating no significant reduction in β-sheet rich amyloid fibril formation. Incubation of HI with Cu-Gly-MOF resulted in a ThT fluorescence intensity of approximately 75% relative to the HI-only control. While this indicates a modest level of inhibition, it is significantly less potent than that observed for *D*-Cu-SMOH (≈30%) and *L*-Cu-SMOH (≈40%).

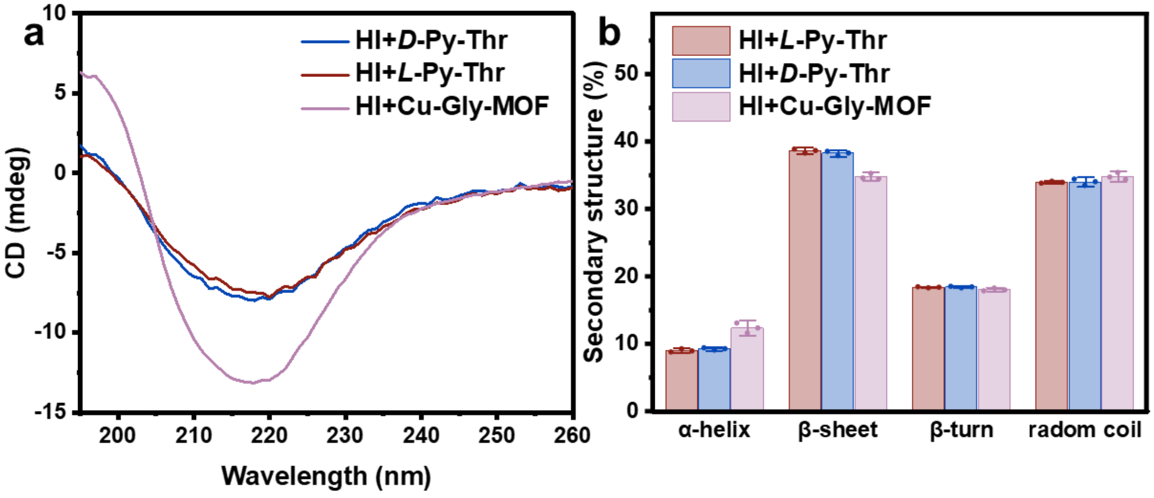

Supplementary Fig. 36. CD spectra and secondary structure analysis of HI with *D*(*L*)-Py-Thr ligands or Cu-Gly-MOF incubation. **a** CD spectra. **b** Secondary structure analysis. Error bars indicate the s.d. (*n* = 3 independent samples). Source data are provided as a Source Data file.

CD spectra of HI incubated with *D*-Py-Thr or *L*-Py-Thr showed the characteristic transition from α-helix to β-sheet structure, similar to the HI control group, with minimal preservation of the native α-helical content. This further confirms the lack of significant inhibitory effect by the free ligands on HI conformational conversion. Analysis of HI secondary structure in the presence of Cu-Gly-MOF showed a slight reduction in β-sheet content compared to the HI-only control, but this effect was much less pronounced than with the *D*(*L*)-Cu-SMOHs.

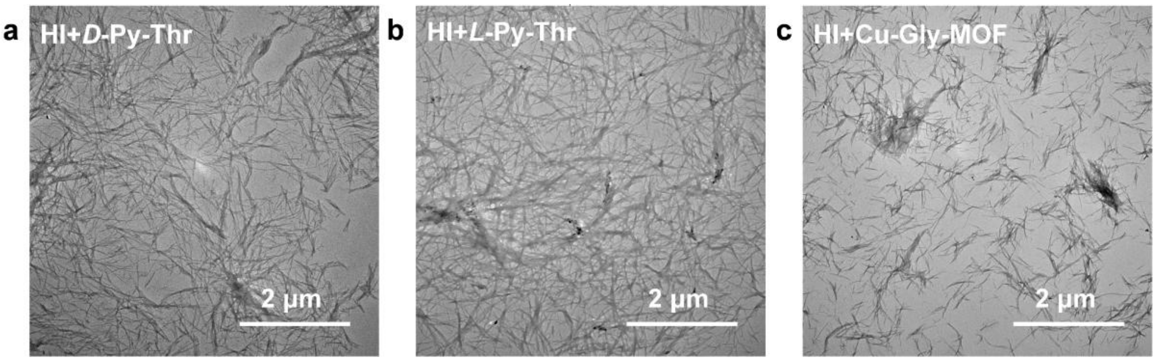

Supplementary Fig. 37. Morphological characterization of HI fibril. TEM imaging of HI with *D*-Py-Thr ligand (**a**), *L*-Py-Thr ligand (**b**), or Cu-Gly-MOF (**c**) incubation.

TEM micrographs (Supplementary Fig. 37a, b) of HI samples incubated with *D*-Py-Thr or *L*-Py-Thr revealed dense networks of mature amyloid fibrils, morphologically indistinguishable from those formed by HI alone (Fig. 4g). TEM micrographs revealed that while some reduction in fibril density was observed in the presence of Cu-Gly-MOF compared to the HI-only control, mature fibrils were still clearly present, albeit perhaps shorter or less bundled (Supplementary Fig. 37c).

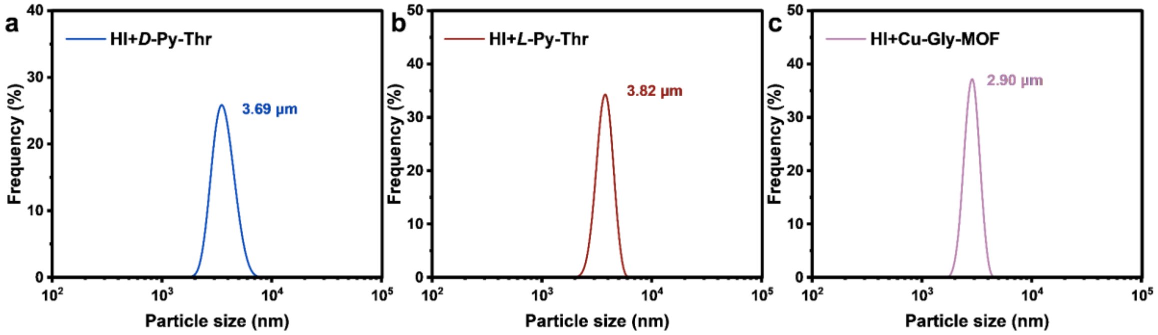

Supplementary Fig. 38. DLS characterization of HI fibril. Size distribution of HI incubated in the presence of *D*-Py-Thr ligand (**a**), *L*-Py-Thr ligand (**b**), or Cu-Gly-MOF (**c**). Source data are provided as a Source Data file.

DLS measurements indicated that the hydrodynamic size of aggregates formed in the presence of *D*-Py-Thr or *L*-Py-Thr (Supplementary Fig. 38a, b) was comparable to that of the HI-only control group (Supplementary Fig. 33), consistent with extensive fibril formation. DLS measurements indicated that the average size of HI aggregates formed in the presence of Cu-Gly-MOF (Supplementary Fig. 38c) was somewhat smaller than that of the HI-only control, consistent with a slight interference in the fibrillization process, but still indicative of substantial aggregation.

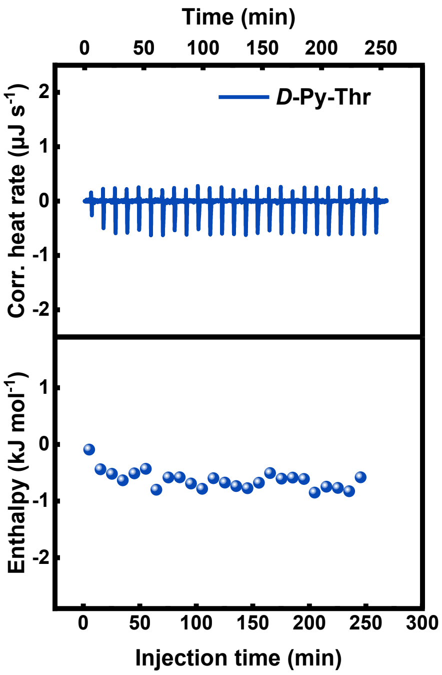

Supplementary Fig. 39. ITC thermograms in the titration of free *D*-Py-Thr ligand into blank buffer at 298.15 K. Source data are provided as a Source Data file.

Intriguingly, *D*-Py-Thr ligand showed negligible calorimetric changes from the blank buffer, indicating that free ligand does not undergo significant self-binding or aggregation at the experimental concentrations.

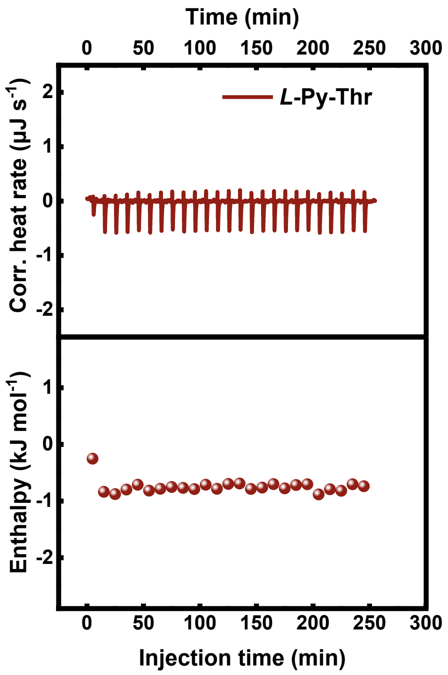

Supplementary Fig. 40. ITC thermograms in the titration of free *L*-Py-Thr ligand into blank buffer at 298.15 K. Source data are provided as a Source Data file.

Similarly, *L*-Py-Thr ligand showed negligible calorimetric changes from the blank buffer, indicating that free ligand does not undergo significant self-binding or aggregation at the experimental concentrations.

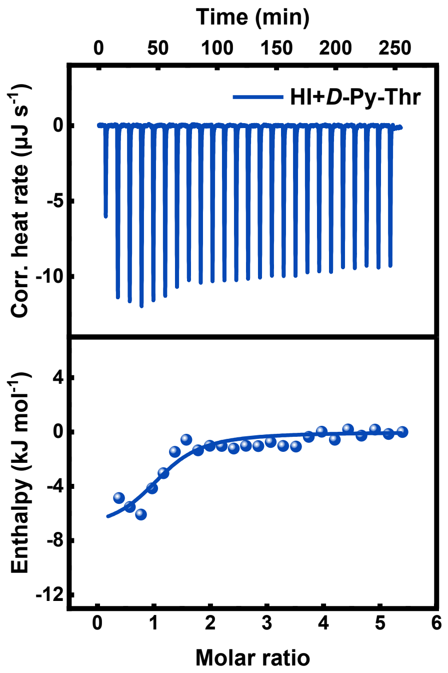

Supplementary Fig. 41. ITC thermograms in the titration of *D*-Py-Thr ligand into HI solution at 298.15 K. Fitting curves are obtained using the unit-point binding model. Source data are provided as a Source Data file.

Intriguingly, the interaction of the free chiral ligand *D*-Py-Thr (hydrochloride) with HI exhibits negative enthalpy change ($\Delta H < 0$) and positive entropy change ($\Delta S > 0$). The negative ΔH suggests that direct binding events, such as electrostatic interactions and/or hydrogen bonding, are exothermic[16,17]. Given that the ligands exist as protonated cations (hydrochloride salts), electrostatic attraction to negatively charged patches on HI at physiological pH is a highly plausible contributor to this exothermic enthalpy. The positive ΔS, while present, was markedly smaller than that observed for the *D*-Cu-SMOHs (Supplementary Table 18).

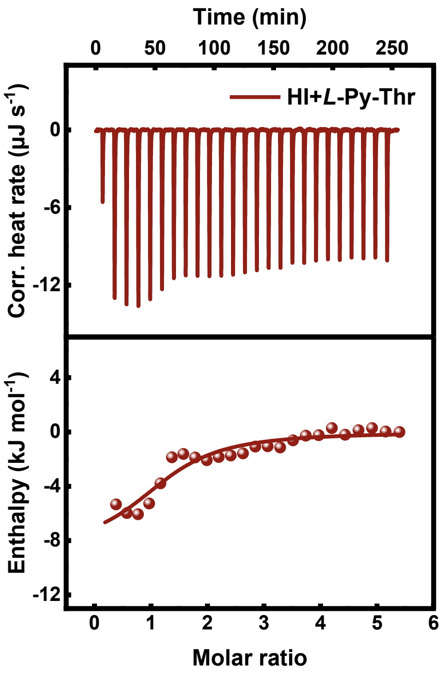

Supplementary Fig. 42. ITC thermograms in the titration of *L*-Py-Thr ligand into HI solution at 298.15 K. Fitting curves are obtained using the unit-point binding model. Source data are provided as a Source Data file.

Similarly, the interaction of the free chiral ligand *L*-Py-Thr (hydrochloride) with HI exhibits negative enthalpy change ($\Delta H < 0$) and positive entropy change ($\Delta S > 0$). The negative ΔH suggests that direct binding events, such as electrostatic interactions and/or hydrogen bonding, are exothermic[16,17]. Given that the ligands exist as protonated cations (hydrochloride salts), electrostatic attraction to negatively charged patches on HI at physiological pH is a highly plausible contributor to this exothermic enthalpy. The positive ΔS, while present, was markedly smaller than that observed for the *L*-Cu-SMOHs (Supplementary Table 18).

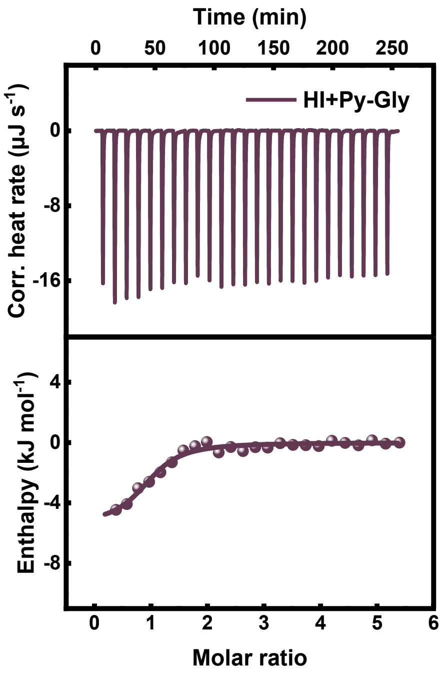

Supplementary Fig. 43. ITC thermograms in the titration of Py-Gly ligand into HI solution at 298.15 K. Fitting curves are obtained using the unit-point binding model. Source data are provided as a Source Data file.

Similar to *D*(*L*)-Py-Thr, the interaction of Py-Gly with HI also yielded $\Delta H < 0$ and $\Delta S > 0$, with the ΔS term again being significantly smaller than that for *D*(*L*)-Cu-SMOHs (Supplementary Table 18). This result reinforces the interpretation that the interaction of protonated free pyridyl ligands with HI is primarily driven by exothermic electrostatic interactions, with a modest entropic contribution[16,17]. The similarity between Py-Gly and *D*(*L*)-Py-Thr in terms of their thermodynamic signature with HI (when free) suggests that the chirality of the threonine side chain in the free ligand does not dramatically alter the primary binding thermodynamics with HI, which seem dominated by the charge.

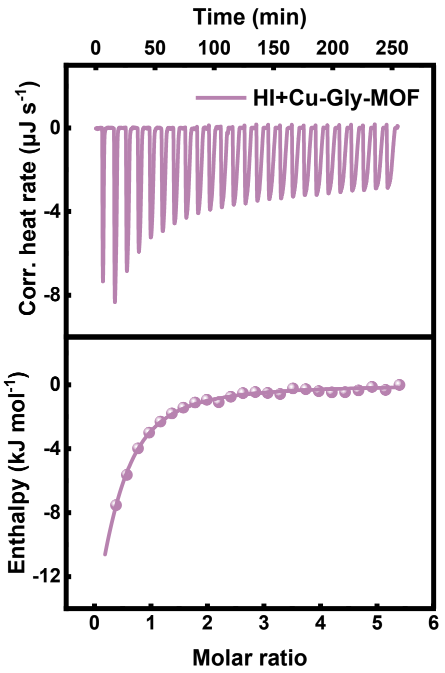

Supplementary Fig. 44. ITC thermograms in the titration of Cu-Gly-MOF into HI solution at 298.15 K. Fitting curves are obtained using the unit-point binding model. Source data are provided as a Source Data file.

We synthesized and performed ITC analysis on an achiral Cu-framework built with a non-aromatic ligand, glycine (Cu-Gly-MOF, Supplementary Fig. 34), interacting with HI. This interaction yielded a negative enthalpy change ($\Delta H < 0$) and, strikingly, a negative entropy change ($\Delta S < 0$). This thermodynamic profile ($\Delta H < 0$, $\Delta S < 0$) is classically indicative of interactions dominated by specific, enthalpically favorable contacts like hydrogen bonding and/or van der Waals interactions, where the formation of a more ordered complex (hence negative ΔS) outweighs any entropy gain from water release[16,17]. This result is in stark contrast to the $\Delta H > 0$, $\Delta S > 0$ profile of the *D*(*L*)-Cu-SMOHs (Supplementary Table 18).

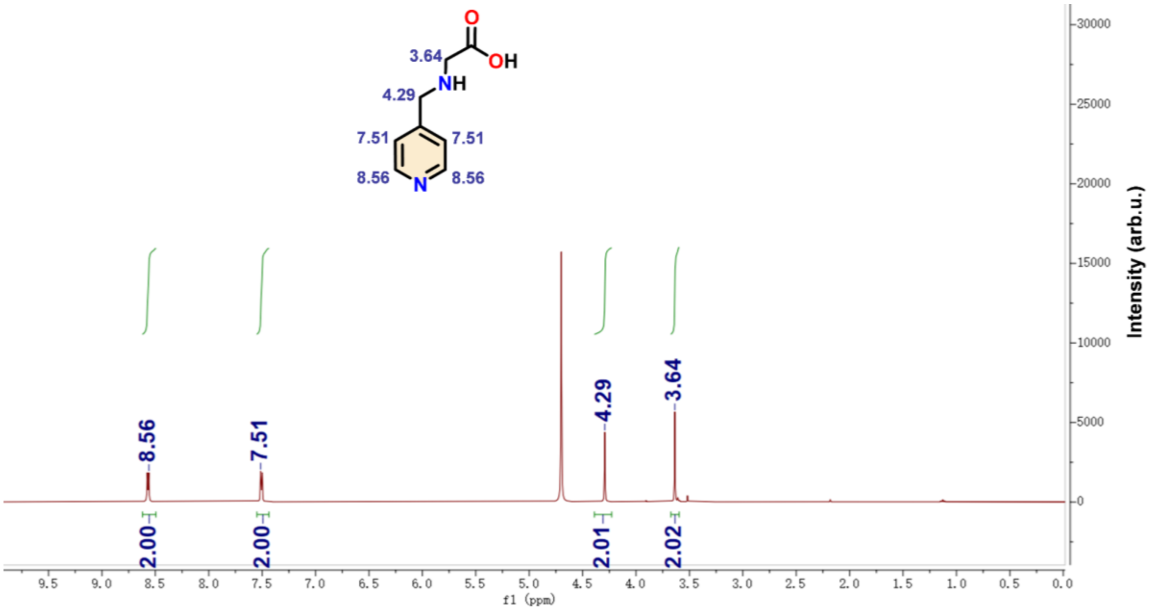

Supplementary Fig. 45. ^{1}H-NMR spectrum of Py-Gly. (The peak centered at 4.70 ppm is the residual solvent peak of D_2O). Peak assignments (ppm): -HN-CH_2 (3.64, s, 2H), -CH_2 (4.29, s, 2H), py-H (7.51, d, 2H), py-H (8.56, d, 2H).

The ^{1}H-NMR spectrum demonstrates the successful synthesis of Py-Gly in high purity.

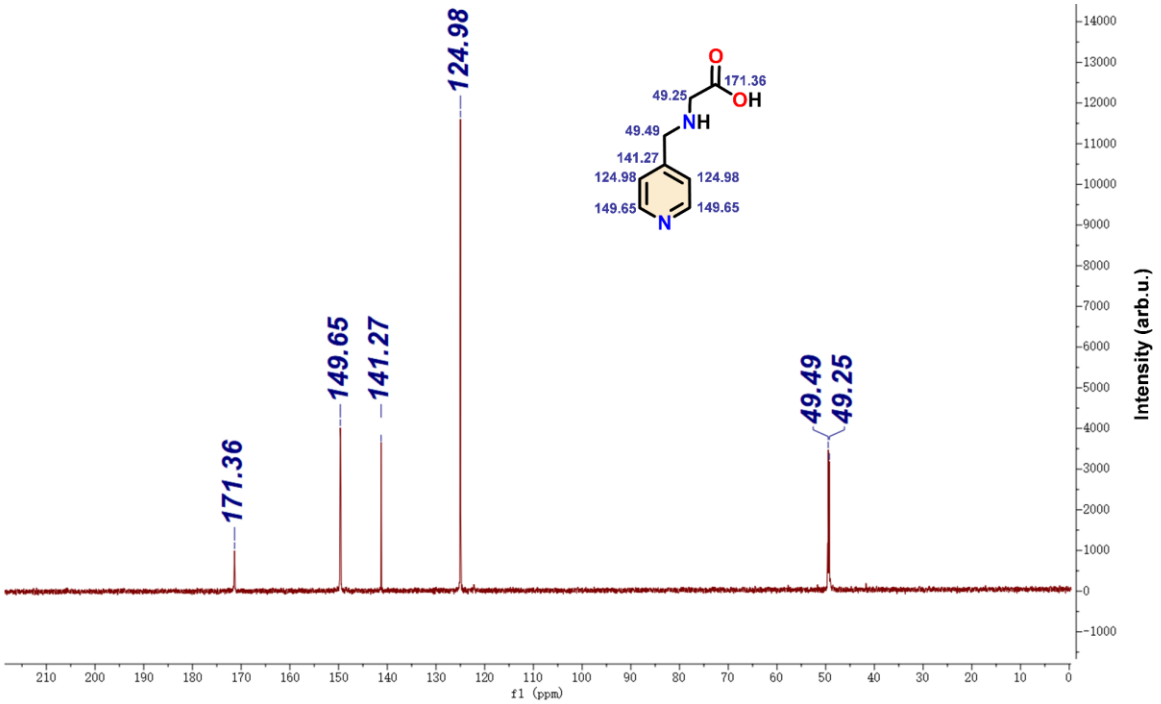

Supplementary Fig. 46. ^{13}C-NMR spectrum of Py-Gly. Peak assignments (ppm): -CH_2 (49.49), -CH_2-HN (49.25), py-C (124.98), py-C (141.27), py-C (149.65), -COOH (171.36).

The ^{13}C-NMR spectrum confirms the successful synthesis of Py-Gly free of impurity.

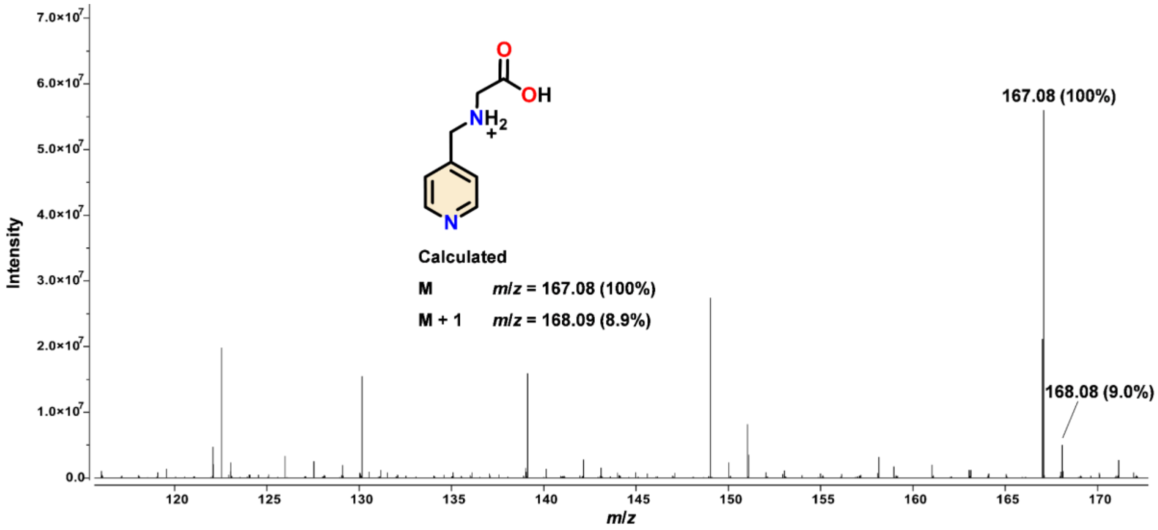

Supplementary Fig. 47. MS spectrum of Py-Gly. Obtained *m*/*z*: 167.08 (100%); *m*/*z*: 168.08 (9.0%). Calculated *m*/*z*: 167.08 (100%); *m*/*z*: 168.09 (8.9%).

As shown above, the peak of *m*/*z* = 167.08 is assigned to the molecular ion peak of protonated Py-Gly and the peak of *m*/*z* = 168.08 is assigned to the isotopic signal. The consistency between observed data with the simulated one further confirm the successful synthesis of Py-Gly ligand.

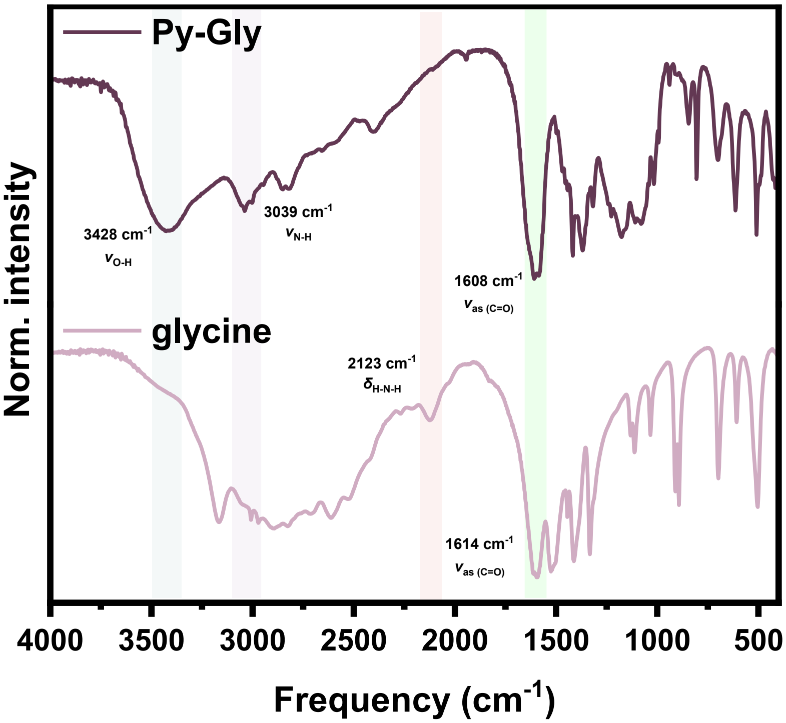

Supplementary Fig. 48. FT-IR spectra of Py-Gly (dark purple curve) raw glycine (light purple curve). Source data are provided as a Source Data file.

The successful formation of Py-Gly ligand is confirmed by the disappearance of the characteristic H-N-H bending vibration at 2123 cm^{-1} and the emergence of a new N-H stretching vibration at 3039 cm^{-1}. The asymmetric stretching vibration of the carboxyl group also undergoes a notable bathochromic shift from 1614 cm^{-1} in the starting glycine to 1608 cm^{-1} in the derived Py-Gly ligand.

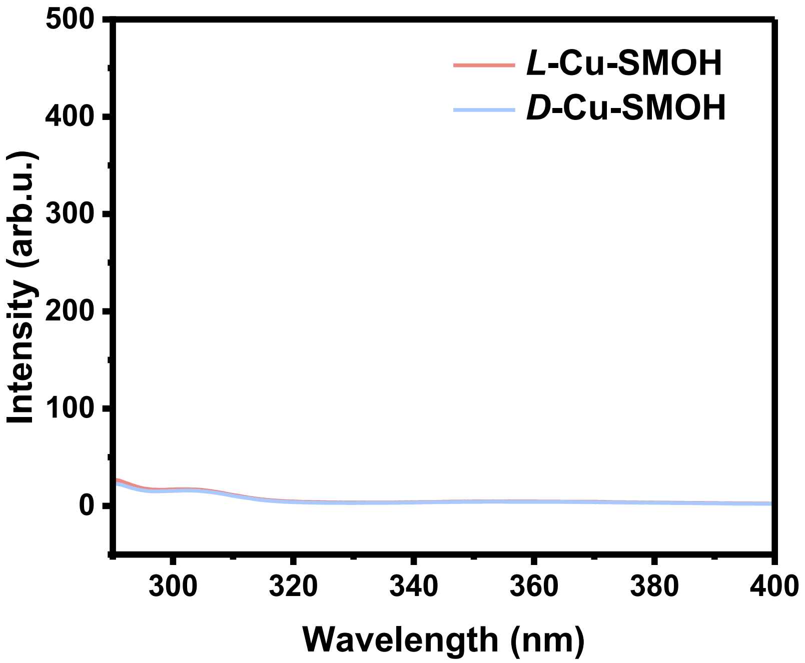

Supplementary Fig. 49. Fluorescence spectra of *D*(*L*)-Cu-SMOHs (excitation wavelength 278 nm). Source data are provided as a Source Data file.

As evidenced by Supplementary Fig. 49, *D*-Cu-SMOH and *L*-Cu-SMOH alone showed almost no fluorescence at the employed experimental conditions.

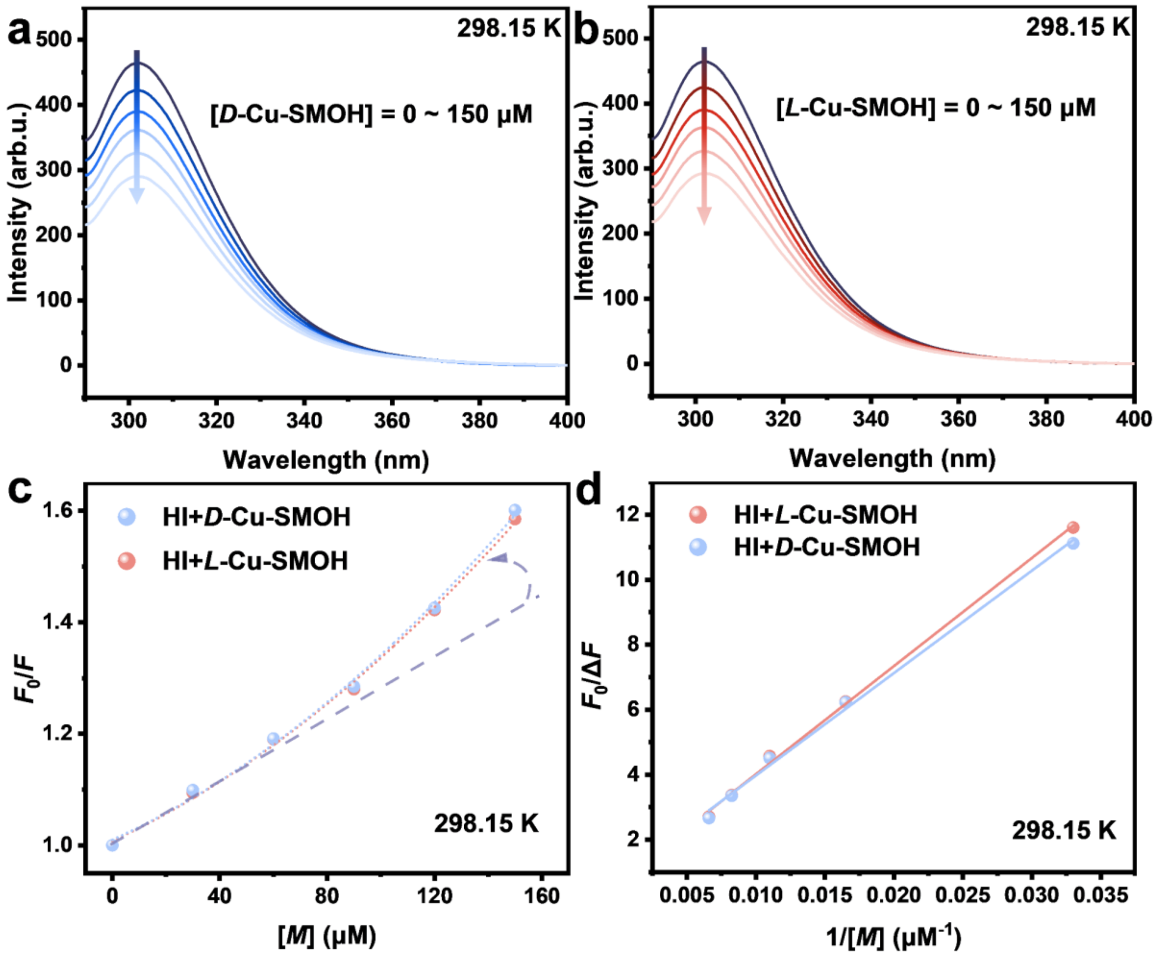

Supplementary Fig. 50. Fluorescence quenching experiment of HI at 298.15 K. Fluorescence quenching spectra of HI by adding *D*-Cu-SMOH (**a**) and *L*-Cu-SMOH (**b**). **c** Stern-Volmer plot. **d** Modified Stern-Volmer plot. The concentration of *D*-Cu-SMOH and *L*-Cu-SMOH is changed from 0 μM to 150 μM. Source data are provided as a Source Data file.

According to the plots shown in Supplementary Fig. 50, the K_a of between HI with *D*-Cu-SMOH and *L*-Cu-SMOH is fitted to be 2.61×10^3 and 2.07×10^3 L mol^{-1}, respectively, at the tested temperature of 298.15 K.

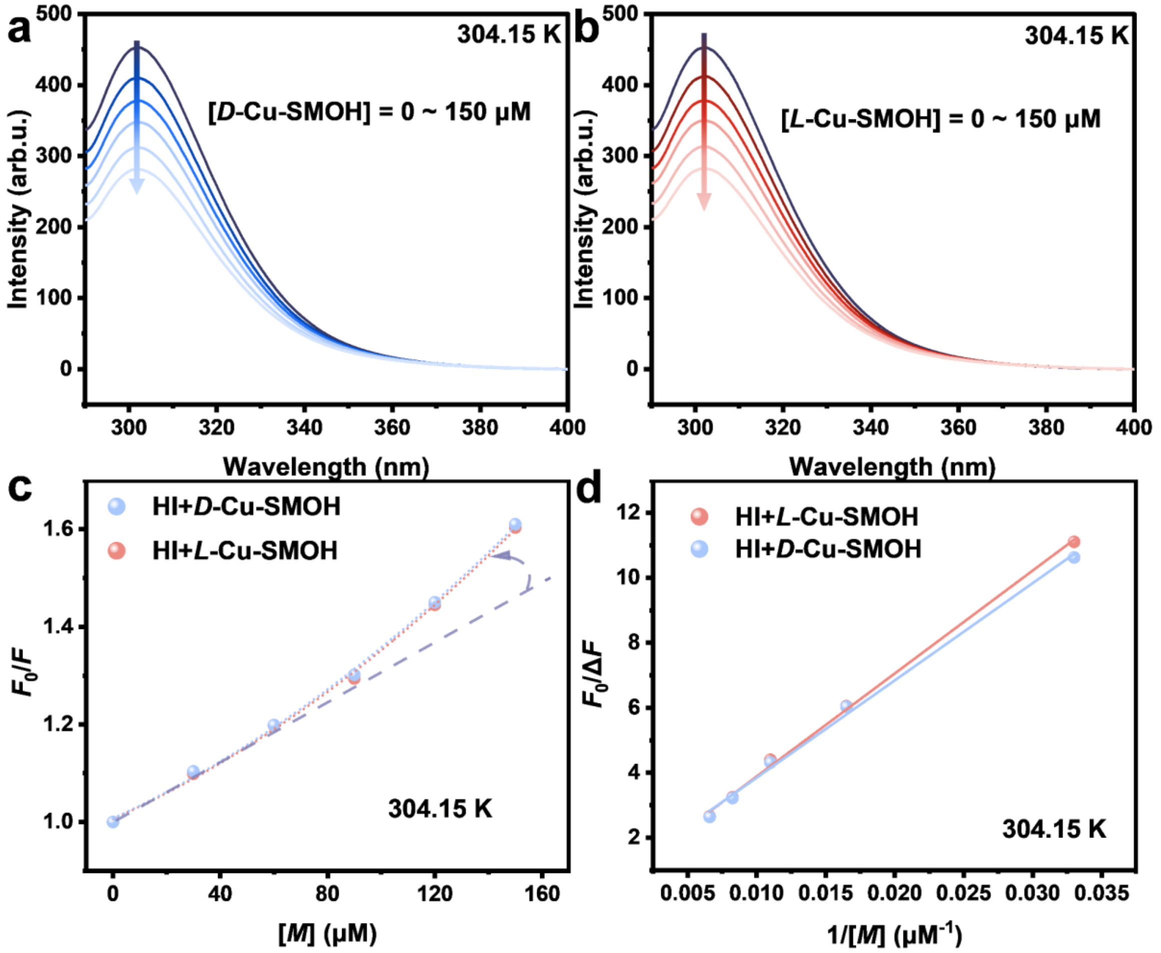

Supplementary Fig. 51. Fluorescence quenching experiment of HI at 304.15 K. Fluorescence quenching spectra of HI by adding *D*-Cu-SMOH (**a**) and *L*-Cu-SMOH (**b**). **c** Stern-Volmer plot. **d** Modified Stern-Volmer plot. The concentration of *D*-Cu-SMOH and *L*-Cu-SMOH is changed from 0 μM to 150 μM. Source data are provided as a Source Data file.

According to the plots shown in Supplementary Fig. 51, the K_a between HI with *D*-Cu-SMOH and *L*-Cu-SMOH is fitted to be 2.85×10^3 and 2.26×10^3 L mol^{-1}, respectively, at the tested temperature of 304.15 K.

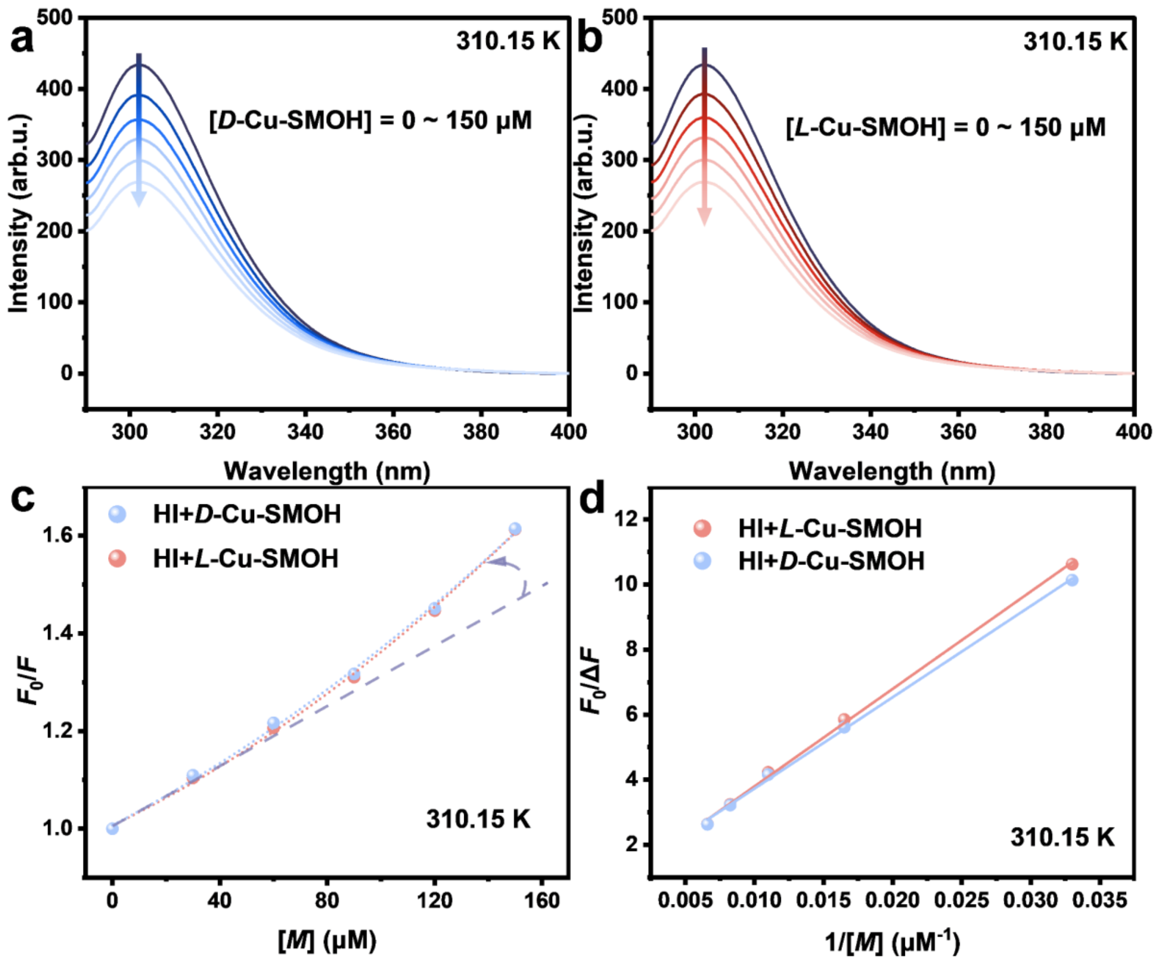

Supplementary Fig. 52. Fluorescence quenching experiment of HI at 310.15 K. Fluorescence quenching spectra of HI by adding *D*-Cu-SMOH (**a**) and *L*-Cu-SMOH (**b**). **c** Stern-Volmer plot. **d** Modified Stern-Volmer plot. The concentration of *D*-Cu-SMOH and *L*-Cu-SMOH is changed from 0 μM to 150 μM. Source data are provided as a Source Data file.

According to the plots shown in Supplementary Fig. 52, the K_a between HI with *D*-Cu-SMOH and *L*-Cu-SMOH is fitted to be 3.28×10^3 and 2.68×10^3 L mol^{-1}, respectively, at the tested temperature of 310.15 K.

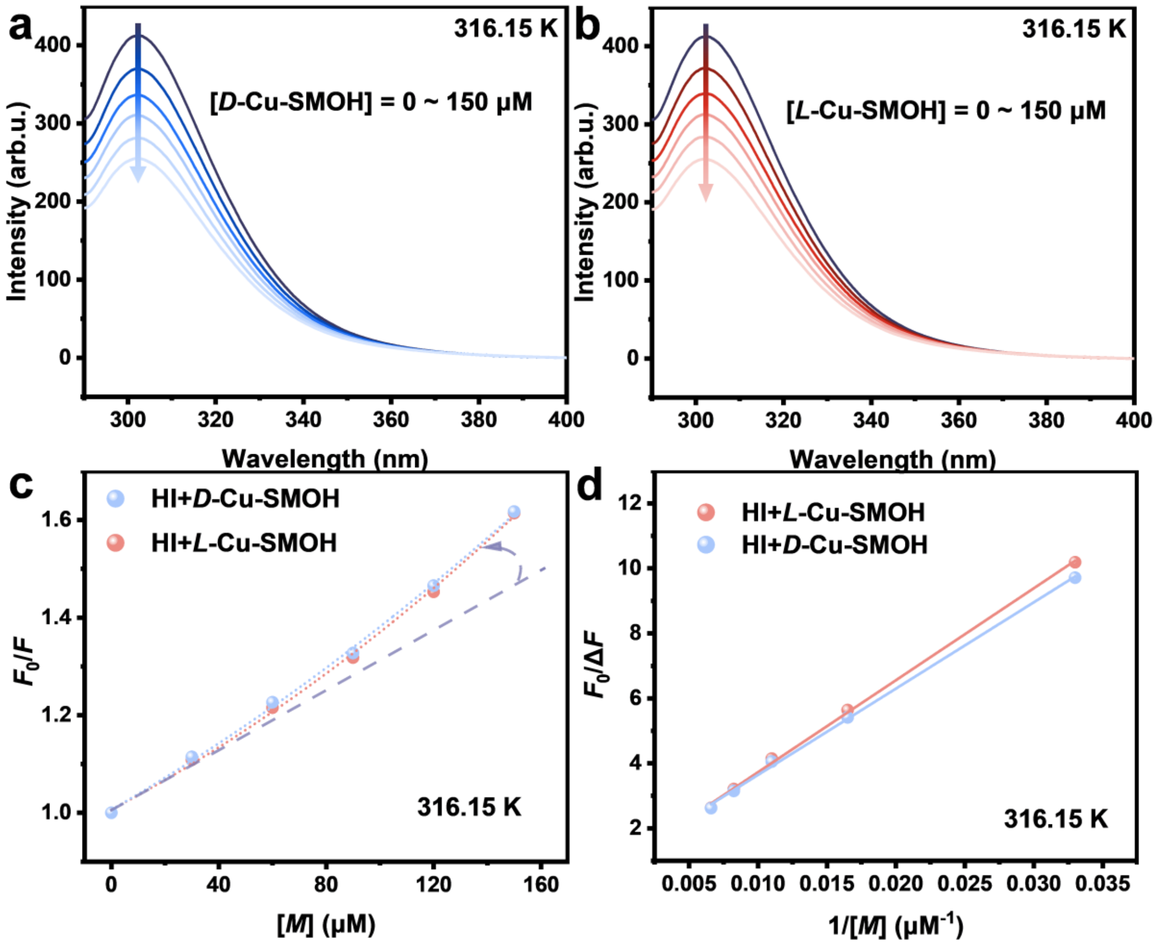

Supplementary Fig. 53. Fluorescence quenching experiment of HI at 316.15 K. Fluorescence quenching spectra of HI by adding *D*-Cu-SMOH (**a**) and *L*-Cu-SMOH (**b**). **c** Stern-Volmer plot. **d** Modified Stern-Volmer plot. The concentration of *D*-Cu-SMOH and *L*-Cu-SMOH is changed from 0 μM to 150 μM. Source data are provided as a Source Data file.

According to the plots shown in Supplementary Fig. 53, the K_a between HI with *D*-Cu-SMOH and *L*-Cu-SMOH is fitted to be 3.72×10^3 and 3.17×10^3 L mol^{-1}, respectively, at the tested temperature of 316.15 K.

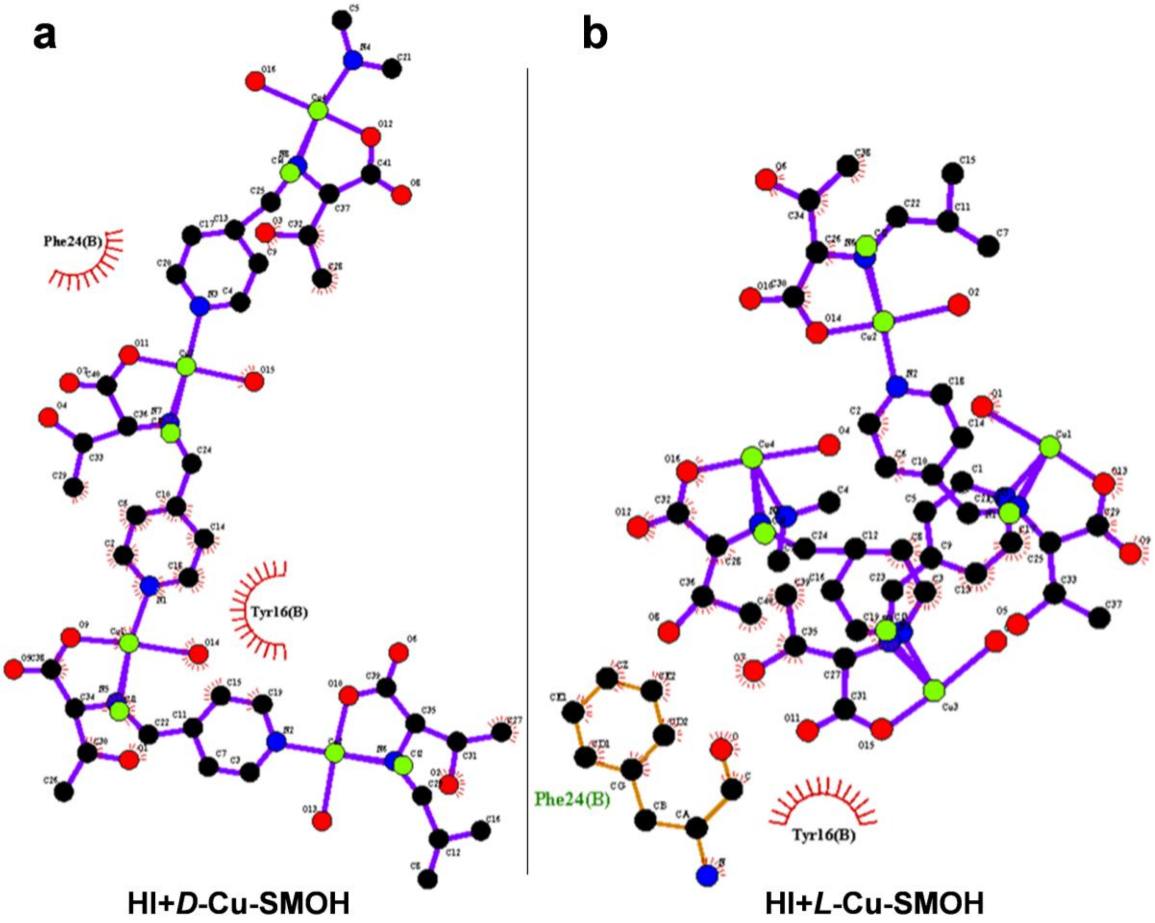

Supplementary Fig. 54. 2D plot of the conformation with the lowest molecular docking energy. a HI with *D*-Cu-SMOH. **b** HI with *L*-Cu-SMOH. AutoDock was used for docking. The red color denotes hydrophobic interactions.

As seen from Supplementary Fig. 54, the outspread *D*-Cu-SMOH exhibits much larger hydrophobic interaction regions available for HI in comparison to *L*-Cu-SMOH.

Supplementary Table 1. The Specific rotation value of *D*-Py-Thr and *L*-Py-Thr.

Sample	***T*** **(ºC)**	***C*** **(mg mL^{-1})**	***L*** **(mm)**	$\boldsymbol{\lambda}$ **(nm)**	**Specific rotation (º)**
D-Py-Thr	30	1	100	589	24.5
L-Py-Thr	30	1	100	589	−25.3
D-Py-Thr + Cu^{2+}	30	1	100	589	35.2
L-Py-Thr + Cu^{2+}	30	1	100	589	−36.1
D-Cu-SMOH	30	1	100	589	51.2
L-Cu-SMOH	30	1	100	589	−50.4

T represents the test temperature, C represents the sample concentration in water, L represents the length of the sample tube, and λ represents the test wavelength of light.

The enantiomeric *D*-Py-Thr and *L*-Py-Thr exhibit specific rotations of $[\alpha] = +24.5°$ and $[\alpha] = -25.3°$, respectively. Upon Cu^{2+} coordination, these values intensify significantly to $[\alpha] = +35.2°$ for the formation of the *D*-Py-Thr-Cu(II) complex and $[\alpha] = -36.1°$ for its *L*-enantiomeric counterpart. The chiroptical amplification trend culminates in the sample of *D*-Cu-SMOH ($[\alpha] = +51.2°$) and *L*-Cu-SMOH ($[\alpha] = -50.4°$), which display nearly twofold enhanced optical activity relative to their constituent ligands as well as the precursor complexes.

Supplementary Table 2. Resolved single-crystal data for *D*-Cu-crystal.

Identification code	*D*-Cu-crystal
CCDC number	2330456
Empirical formula	$C_{10}H_{15}ClCuN_2O_4$
Formula weight	326.23
Temperature/K	100.1(3)
Crystal system	tetragonal
Space group	$P4_32_12$
a/Å	9.12160(10)
b/Å	9.12160(10)
c/Å	35.5635(3)
α/°	90
β/°	90
γ/°	90
Volume/Å^3	2959.01(7)
Z	8
ρ_{calc}/g·cm^{-3}	1.465
μ/mm^{-1}	3.834
F(000)	1336.0
Crystal size/mm^3	0.14 × 0.12 × 0.1
Radiation	Cu K_α (λ = 1.54184)
2θ range for data collection/°	9.948 to 148.748
Index ranges	$-9 \le h \le 11$, $-10 \le k \le 7$, $-42 \le l \le 43$
Reflections collected	13855
Independent reflections	2947 [R_{int} = 0.0252, R_{sigma} = 0.0190]
Data/restraints/parameters	2947/0/166
Goodness-of-fit on F^2	1.075

Final R indexes [$I \geq 2\sigma$ (I)]	R_1 = 0.0294, wR_2 = 0.0734
Final R indexes [all data]	R_1 = 0.0299, wR_2 = 0.0736
Largest diff. peak/hole / e Å^{-3}	0.62/−0.46
Flack parameter	0.002(8)

Supplementary Table 3. Fractional atomic coordinates ($\times10^4$) and equivalent isotropic displacement arameters ($Å^2\times10^3$) for *D*-Cu-crystal. U_{eq} is defined as 1/3 of the trace of the orthogonalised U_{IJ} tensor.

**Atom**	***x***	***y***	***z***	**U(eq)**
Cu1	398.2(5)	4127.4(5)	5962.3(2)	13.21(13)
Cl1	−2042.3(8)	4164.8(10)	5824.6(2)	19.63(18)
O1	659(3)	7177(3)	6141.7(6)	22.4(5)
O2	3819(2)	5432(3)	6565.1(6)	16.0(5)
O3	2455(3)	4389(3)	6121.5(6)	15.1(5)
O4	937(3)	1667(2)	5973.1(7)	17.4(5)
N1	−796(3)	4054(3)	7920.1(7)	14.1(5)
N2	−7(3)	4569(3)	6512.4(7)	13.6(5)
C2	363(4)	3295(4)	7796.0(8)	18.7(7)
C3	576(4)	3000(4)	7416.2(9)	18.0(7)
C4	−430(4)	3492(4)	7155.8(8)	13.6(6)
C5	−1648(4)	4268(4)	7287.5(9)	15.9(7)
C6	−1777(4)	4542(4)	7668.8(9)	16.4(6)
C7	−193(4)	3215(4)	6741.2(8)	15.9(7)
C8	−373(4)	7768(4)	6758.7(10)	23.5(8)
C9	899(4)	7133(4)	6539.9(9)	18.0(6)
C10	1220(3)	5521(4)	6635.7(8)	13.6(7)
C11	2605(4)	5064(3)	6428.5(9)	13.2(6)

Supplementary Table 4. Hydrogen atom coordinates (Å×10^4) and isotropic displacement parameters (Å^2×10^3) for *D*-Cu-crystal.

Atom	*x*	*y*	*z*	U(eq)
H1	10.29	7801.17	6092.21	34
H4A	146.9	1177.62	6000.89	26
H4B	1439.39	1468.06	6169.11	26
H2	−934.78	5149.11	6527.97	16
H2A	1061.72	2947.72	7972.79	22
H3	1411.5	2461.39	7336.48	22
H5	−2377.94	4602.54	7117.39	19
H6	−2590.75	5096.43	7755.76	20
H7A	689.31	2595.01	6710.21	19
H7B	−1041.19	2658.7	6642.28	19
H8A	−449.5	8820.48	6706.97	35
H8B	−210.18	7615.79	7028.3	35
H8C	−1282.38	7279.22	6682.74	35
H9	1793.78	7724.16	6598.04	22
H10	1370.1	5414.66	6912.95	16

Supplementary Table 5. Bond lengths for *D*-Cu-crystal.

Atom	Atom	Length/Å	Atom	Atom	Length/Å
Cu1	Cl1	2.2796(9)	N2	C7	1.488(4)
Cu1	O3	1.974(2)	N2	C10	1.483(4)
Cu1	O4	2.298(2)	C2	C3	1.391(4)
Cu1	N1[1]	1.993(3)	C3	C4	1.379(5)
Cu1	N2	2.031(3)	C4	C5	1.398(5)
O1	C9	1.433(4)	C4	C7	1.511(4)
O2	C11	1.255(4)	C5	C6	1.384(4)
O3	C11	1.261(4)	C8	C9	1.512(5)
N1	C2	1.338(4)	C9	C10	1.538(5)
N1	C6	1.341(4)	C10	C11	1.521(4)

[1] 1/2 − *Y*, 1/2 + *X*, -1/4 + *Z*

Supplementary Table 6. Bond angles for *D*-Cu-crystal.

Atom	Atom	Atom	Angle/°	Atom	Atom	Atom	Angle/°
Cl1	Cu1	O4	103.13(7)	N1	C2	C3	122.1(3)
O3	Cu1	Cl1	171.07(7)	C4	C3	C2	119.7(3)
O3	Cu1	O4	84.82(9)	C3	C4	C5	117.9(3)
O3	Cu1	N1[1]	91.99(10)	C3	C4	C7	120.4(3)
O3	Cu1	N2	82.69(10)	C5	C4	C7	121.7(3)
N1[1]	Cu1	Cl1	92.11(8)	C6	C5	C4	119.2(3)
N1[1]	Cu1	O4	89.81(10)	N1	C6	C5	122.4(3)
N1[1]	Cu1	N2	166.03(11)	N2	C7	C4	114.3(3)
N2	Cu1	Cl1	91.51(8)	O1	C9	C8	112.4(3)
N2	Cu1	O4	102.50(10)	O1	C9	C10	105.9(3)
C11	O3	Cu1	114.2(2)	C8	C9	C10	113.5(3)
C2	N1	Cu1[2]	118.5(2)	N2	C10	C9	110.5(3)
C2	N1	C6	118.6(3)	N2	C10	C11	108.8(2)
C6	N1	Cu1[2]	122.6(2)	C11	C10	C9	108.3(3)
C7	N2	Cu1	112.52(19)	O2	C11	O3	124.1(3)
C10	N2	Cu1	105.29(18)	O2	C11	C10	118.2(3)
C10	N2	C7	114.2(2)	O3	C11	C10	117.6(3)

[1] 1/2 − *Y*, 1/2 + *X*, −1/4 + *Z*;

[2] -1/2 + *Y*, 1/2 − *X*, 1/4 + *Z*

Supplementary Table 7. Hydrogen bonds for *D*-Cu-crystal [Å and °].

D[1]-H...X[2]	d(D-H)	d(H...X)	d(D...X)	<(DHX)
O1-H1...O2	0.840(3)	2.023(3)	2.849(4)	167.6(2)
O4-H4A...O2	0.853(3)	1.918(3)	2.723(4)	156.86(17)

"D" stands for hydrogen-donor atom;

"X" stands for hydrogen-acceptor atom.

571 **Supplementary Table 8.** Crystal data and structure refinement for *L*-Cu-crystal.

Identification code	*L*-Cu-crystal
CCDC number	2330457
Empirical formula	$C_{10}H_{15}ClCuN_2O_4$
Formula weight	326.23
Temperature/K	169.99(10)
Crystal system	tetragonal
Space group	$P4_12_12$
a/Å	9.13620(10)
b/Å	9.13620(10)
c/Å	35.5787(6)
α/°	90
β/°	90
γ/°	90
Volume/Å^3	2969.76(8)
Z	8
ρ_{calc}/g·cm^3	1.459
μ/mm^{-1}	3.820
F(000)	1336.0
Crystal size/mm^3	0.14 × 0.12 × 0.09
Radiation	Cu K_α (λ = 1.54184)
2θ range for data collection/°	9.944 to 147.692
Index ranges	$-11 \leq h \leq 11$, $-11 \leq k \leq 10$, $-44 \leq l \leq 40$
Reflections collected	20830
Independent reflections	2967 [R_{int} = 0.0392, R_{sigma} = 0.0243]
Data/restraints/parameters	2967/0/166
Goodness-of-fit on F^2	1.067

Final R indexes [$I \geq 2\sigma$ (I)]	R_1 = 0.0297, wR_2 = 0.0754
Final R indexes [all data]	R_1 = 0.0316, wR_2 = 0.0761
Largest diff. peak/hole / e Å^{-3}	0.45/−0.48
Flack parameter	0.006(9)

Supplementary Table 9. Fractional atomic coordinates (×10^4) and equivalent isotropic displacement parameters (Å^2×10^3) for *L*-Cu-crystal. U_{eq} is defined as 1/3 of the trace of the orthogonalised U_{IJ} tensor.

**Atom**	***x***	***y***	***z***	**U(eq)**
Cu1	−875.3(5)	5361.7(5)	5959.8(2)	18.06(13)
Cl1	−850.5(11)	2931.5(8)	5822.1(2)	28.64(19)
O1	−3343(2)	5906(3)	5972.6(7)	24.3(5)
O2	612(3)	2825(3)	3640.2(7)	31.3(6)
O3	3779(2)	4572(2)	4061.0(6)	21.2(5)
O4	2416(2)	5608(2)	3617.8(6)	18.3(4)
N1	−801(3)	5909(3)	5418.9(7)	18.9(5)
N2	−43(3)	5432(3)	4007.7(7)	16.7(5)
C1	355(4)	6665(4)	5291.9(9)	26.8(7)
C2	559(4)	6966(4)	4913.3(9)	25.7(7)
C3	−452(4)	6488(3)	4652.8(8)	18.9(6)
C4	−1664(3)	5722(4)	4786.8(9)	23.3(7)
C5	−1790(4)	5446(4)	5166.8(9)	22.7(7)
C6	−221(4)	6777(3)	4239.8(8)	19.9(7)
C7	1178(3)	4478(3)	4131.1(8)	17.6(6)
C8	2567(3)	4938(3)	3924.6(9)	17.7(6)
C9	866(4)	2869(3)	4036.2(9)	23.2(6)
C10	−395(5)	2240(4)	4258.1(11)	33.8(9)

Supplementary Table 10. Hydrogen atom coordinates (Å×10^4) and isotropic displacement parameters (Å^2×10^3) for *L*-Cu-crystal.

**Atom**	***x***	***y***	***z***	**U(eq)**
H1A	−3830.28	5122.87	6006.9	36
H1B	−3529.39	6426.92	6165.02	36
H2	−63.48	2261.31	3594.92	47
H2A	−952.48	4866.51	4022.5	20
H1	1045.44	6999	5463.73	32
H2B	1376.99	7491.03	4835.28	31
H4	−2383.13	5399.81	4621.23	28
H5	−2594.08	4916.05	5251.67	27
H6A	−1050.51	7327.6	4145.08	24
H6B	643.55	7382.29	4209.97	24
H7	1322.02	4582.89	4402.75	21
H9	1745.36	2294.18	4091.57	28
H10A	−1286.22	2720.94	4186.17	51
H10B	−224.62	2387.66	4521.6	51
H10C	−475.76	1210.79	4207.5	51

Supplementary Table 11. Bond lengths for *L*-Cu-crystal.

Atom	Atom	Length/Å	Atom	Atom	Length/Å
Cu1	Cl1	2.2739(9)	N2	C6	1.490(4)
Cu1	O1	2.309(2)	N2	C7	1.482(4)
Cu1	O4[1]	1.974(2)	C1	C2	1.387(5)
Cu1	N1	1.989(2)	C2	C3	1.380(5)
Cu1	N2[1]	2.025(2)	C3	C4	1.394(5)
O2	C9	1.428(4)	C3	C6	1.508(4)
O3	C8	1.254(4)	C4	C5	1.380(5)
O4	C8	1.259(4)	C7	C8	1.526(4)
N1	C1	1.340(4)	C7	C9	1.535(4)
N1	C5	1.342(4)	C9	C10	1.510(5)

[1] $1/2 - Y$, $1/2 + X$, $1/4 + Z$

Supplementary Table 12. Bond angles for *L*-Cu-crystal.

Atom	Atom	Atom	Angle/°	Atom	Atom	Atom	Angle/°
Cl1	Cu1	O1	102.96(7)	N1	C1	C2	122.4(3)
O4[1]	Cu1	Cl1	171.24(7)	C3	C2	C1	120.0(3)
O4[1]	Cu1	O1	84.87(9)	C2	C3	C4	117.5(3)
O4[1]	Cu1	N1	91.85(10)	C2	C3	C6	120.4(3)
O4[1]	Cu1	N2[1]	82.81(9)	C4	C3	C6	122.2(3)
N1	Cu1	Cl1	92.09(8)	C5	C4	C3	119.5(3)
N1	Cu1	O1	89.91(10)	N1	C5	C4	122.8(3)
N1	Cu1	N2[1]	165.97(11)	N2	C6	C3	114.3(3)
N2[1]	Cu1	Cl1	91.56(8)	N2	C7	C8	108.7(2)
N2[1]	Cu1	O1	102.46(10)	N2	C7	C9	111.0(3)
C8	O4	Cu1[2]	114.3(2)	C8	C7	C9	108.2(2)
C1	N1	Cu1	118.9(2)	O3	C8	O4	124.2(3)
C1	N1	C5	117.9(3)	O3	C8	C7	118.3(3)
C5	N1	Cu1	123.0(2)	O4	C8	C7	117.4(3)
C6	N2	Cu1[2]	112.87(19)	O2	C9	C7	105.9(2)
C7	N2	Cu1[2]	105.41(18)	O2	C9	C10	112.4(3)
C7	N2	C6	113.8(2)	C10	C9	C7	113.0(3)

[1] 1/2 − *Y*, 1/2 + *X*, 1/4 + *Z*;

[2] -1/2 + *Y*, 1/2 − *X*, −1/4 + *Z*

Supplementary Table S13. Hydrogen bonds for *L*-Cu-crystal [Å and °].

D[1]-H...X[2]	d(D-H)	d(H...X)	d(D...X)	<(DHX)
O2-H2...O3	0.820(3)	2.0571(19)	2.848(4)	162.1(2)
O1-H1A...O3	0.851(3)	1.9226(19)	2.724(3)	156.28(17)

"D" stands for hydrogen-donor atom;

"X" stands for hydrogen-acceptor atom.

Supplementary Table 14. Functional groups assignments for *D*(*L*)-SMOHs.

Band	IR (cm^{-1})	Assignment
v_1	1641	C=O antisymmetric stretching of COO-
v_2	1620	C=N stretching of Py ring
v_3	1595	C=C stretching of Py ring
v_4	1562	C=C stretching of Py ring
v_5	1430	C=O symmetric stretching of COO-
v_6	1392	C=C of Py ring
v_7	1317	C–O stretching of COO-
v_8	1220	C–H stretching of Py ring

As presented in Supplementary Table 14, the signals at 1641 (v_1), 1430 (v_5), and 1317 (v_7) cm^{-1} for the enantiomers *D*-Cu-SMOH and *L*-Cu-SMOH are attributed to the C=O and C–O stretching vibrations originating from the carboxylate (COO^-) moiety, while other characteristic signals are generated by aromatic pyridine C=C/C=N bond vibrations (v_2, v_3, v_4, and v_6) and pyridine ring C–H vibrations (v_8)[4-7].

Supplementary Table 15. All integrals of $3d$ orbits $\langle n|\hat{\mathrm{L}}_i|0\rangle$ ($i = x, y, z$).

	$\langle \boldsymbol{d_{xy}} \|$	$\langle \boldsymbol{d_{yz}} \|$	$\langle \boldsymbol{d_{xz}} \|$	$\langle \boldsymbol{d_{x^2-y^2}} \|$	$\langle \boldsymbol{d_{z^2}} \|$
$\hat{\mathbf{L}}_x \| \boldsymbol{d_{xy}} \rangle$	0	0	i	0	0
$\hat{\mathbf{L}}_x \| \boldsymbol{d_{yz}} \rangle$	0	0	0	i	$\sqrt{3}$i
$\hat{\mathbf{L}}_x \| \boldsymbol{d_{xz}} \rangle$	−i	0	0	0	0
$\hat{\mathbf{L}}_x \| \boldsymbol{d_{x^2-y^2}} \rangle$	0	−i	0	0	0
$\hat{\mathbf{L}}_x \| \boldsymbol{d_{z^2}} \rangle$	0	$-\sqrt{3}$i	0	0	0
$\hat{\mathbf{L}}_y \| \boldsymbol{d_{xy}} \rangle$	0	−i	0	0	0
$\hat{\mathbf{L}}_y \| \boldsymbol{d_{yz}} \rangle$	i	0	0	0	0
$\hat{\mathbf{L}}_y \| \boldsymbol{d_{xz}} \rangle$	0	0	0	i	$-\sqrt{3}$i
$\hat{\mathbf{L}}_y \| \boldsymbol{d_{x^2-y^2}} \rangle$	0	0	−i	0	0
$\hat{\mathbf{L}}_y \| \boldsymbol{d_{z^2}} \rangle$	0	0	$\sqrt{3}$i	0	0
$\hat{\mathbf{L}}_z \| \boldsymbol{d_{xy}} \rangle$	0	0	0	−2i	0
$\hat{\mathbf{L}}_z \| \boldsymbol{d_{yz}} \rangle$	0	0	−i	0	0
$\hat{\mathbf{L}}_z \| \boldsymbol{d_{xz}} \rangle$	0	i	0	0	0
$\hat{\mathbf{L}}_z \| \boldsymbol{d_{x^2-y^2}} \rangle$	2i	0	0	0	0
$\hat{\mathbf{L}}_z \| \boldsymbol{d_{z^2}} \rangle$	0	0	0	0	0

These values respectively these values correspond to the numbers on each arrow of the magic pentagon (Supplementary Fig. 22).

Supplementary Table 16. EXAFS fitting parameters at the Cu *K*-edge for various samples.

Sample	Path	N^a	R (Å)^b	σ^2 (Å^2)^c	ΔE_0 (eV)^d	R factor
D-Cu-crystal	Cu-O/N	3.7	1.99(8)	0.009(8)	5.30(3)	0.017
	Cu-Cl	1.0	2.28(5)	0.011(8)	5.30(3)	
D-Cu-SMOH	Cu-O/N	4.6	1.95(2)	0.009(5)	1.75(9)	0.018
	Cu-Cl	1.0	2.25(0)	0.010(5)	1.75(9)	

$^a N$: coordination numbers, $^b R$: bond distance, $^c \sigma^2$: Debye-Waller factors, $^d \Delta E_0$: the inner potential correction, R factor: goodness of fit.

EXAFS analysis of *D*-Cu-crystal reveals a Cu-O/N coordination number of 3.7, closely matching the value of 4 derived from single-crystal X-ray diffraction (SXRD). Notably, disassembled *D*-Cu-SMOH induces a marked increase in coordination number to 4.6, attributable to the incorporation of one additional water molecule into the Cu coordination shell.

Supplementary Table 17. Effect of *D*-Cu-SMOH and *L*-Cu-SMOH on the kinetic parameters of HI fibrillation.

Sample	$t_{1/2}$ (min)	t_{lag} (min)	τ (min)	υ (min^{-1})	FL
HI	150.67	125.09	12.79	0.078	100%
HI+*L*-Cu-SMOH	159.62	132.78	13.42	0.075	40%
HI+*D*-Cu-SMOH	168.18	135.30	16.44	0.061	30%

The HI fibrillation kinetics were modeled by approximating the growth phase as a quasi-primary process, where ThT fluorescence intensity exhibits a linear correlation with the amyloid aggregate amount. The lag time (t_{lag}) and the growth rate (v) can be obtained by fitting the ThT kinetic data using the Sigmoidal profile [Supplementary Eq. (2)-(4)][18]:

$$F = F_0 + \frac{F_{max} - F_0}{1 + e^{-\frac{t - t_{1/2}}{\tau}}} \quad (2)$$

$$t_{lag} = t_{1/2} - 2\tau \quad (3)$$

$$\upsilon = 1/\tau \quad (4)$$

Here, F, F_0 and $F_{\max}$ represent instantaneous, initial, and equilibrium ThT fluorescence intensities, respectively. The parameter $t_{1/2}$ indicates the characteristic time at FL intensity reaching half of $F_{\max}$, τ signifies the growth rate constant, υ represents the growth rate, and t_{lag} denotes the lag time.

Compared to the HI-only control, *D*-Cu-SMOH and *L*-Cu-SMOH suppress amyloidogenesis by 30% and 40%, respectively, according to FL intensity.

Supplementary Table 18. Interactions thermodynamics between HI at 298.15 K.

Sample	ΔH (kJ mol^{-1})	ΔS (J mol^{-1} K^{-1})	TΔS (kJ mol^{-1})	ΔG (kJ mol^{-1})
HI+*D*-Cu-SMOH	5.56	118.20	35.24	−29.68
HI+*L*-Cu-SMOH	7.21	113.57	33.86	−26.65
HI+*D*-Py-Thr	−7.29	62.74	18.71	−26
HI+*L*-Py-Thr	−8.61	52.47	15.64	−24.25
HI+Py-Gly	−5.44	72.18	21.52	−26.96
HI+Cu-Gly-MOF	−35.77	−42.37	−12.63	−23.14

As shown in Supplementary Table 18, the interaction of *D*(*L*)-Cu-SMOHs with HI is characterized by ΔH > 0 and ΔS >> 0, dominated by hydrophobic π-π interactions[16,17]. In contrast, *D*(*L*)-Py-Thr interacts with HI exhibiting ΔH < 0 and a smaller positive ΔS, primarily driven by electrostatic forces. The Cu-Gly-MOF with HI interaction further differs, showing ΔH < 0 and ΔS < 0, indicative of dominant hydrogen bonding.

Supplementary Table 19. Fitting results of modified SV equation and van't Hoff equation at different temperatures (298.15, 304.15, 310.15, 316.15 K).

Sample	***T***	K_a	**Δ*H***	**Δ*S***	**Δ*G***
	K	10^3 L mol^{-1}	kJ mol^{-1}	J mol^{-1} K^{-1}	kJ mol^{-1}
D-Cu-SMOH	298.15	2.61 ± 0.70	14.73 ± 1.47	114.82 ± 4.80	−19.51
	304.15	2.85 ± 0.69			−20.20
	310.15	3.28 ± 0.44			−20.89
	316.15	3.72 ± 0.43			−21.58
L-Cu-SMOH	298.15	2.07 ± 0.49	16.69 ± 1.66	119.39 ± 5.40	−18.89
	304.15	2.26 ± 0.49			−19.61
	310.15	2.68 ± 0.42			−20.33
	316.15	3.17 ± 0.42			−21.04

According to the fitting results of the modified SV equation[19] at different temperatures, we calculate corresponding thermodynamic parameters based on van't Hoff equation[17] in Supplementary Table 19. Compared to its *L*-counterpart, *D*-Cu-SMOH exhibits stronger binding affinities (K_a) as well as lower Gibbs free energies (ΔG) across all investigated temperatures.

Supplementary Table 20. Binding energies and binding sites for molecular docking.

Conformer	$E_{Binding}$ (kJ mol^{-1})	Bonding Sites
L-Cu-SMOH (First lowest-energy)	−8.74	Y16B, F24B, F25B, Y26B
L-Cu-SMOH (Second lowest-energy)	−4.85	Y19A, F25B
L-Cu-SMOH (Third lowest-energy)	−4.69	F24B, F25B, Y26B
D-Cu-SMOH (First lowest-energy)	−10.25	Y16B, F24B, F25B, Y26B
D-Cu-SMOH (Second lowest-energy)	−7.57	S9B, H10B
D-Cu-SMOH (Third lowest-energy)	−6.74	Y19A, F25B, Y26B

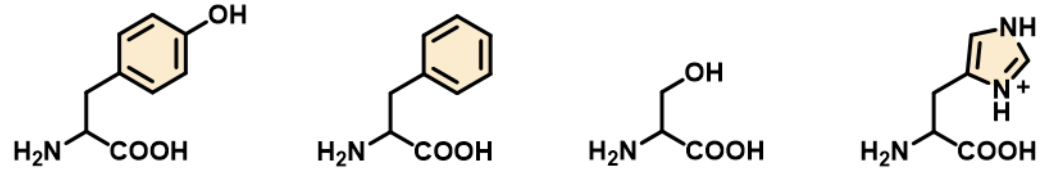

Tyrosine(Tyr, Y) Phenylalanine(Phe, F) Serine(Ser, S) Histidine(His, H)

Molecular docking simulations reveal distinct enantiomeric discrimination during HI binding, with the *D*-Cu-SMOH demonstrating both superior thermodynamic favorability and enhanced conformational stability compared to its *L*-enantiomer. In detail, the three most thermodynamically stable conformers (Supplementary Table 20) show that *D*-Cu-SMOH displays significantly stronger binding affinities with energies of −10.26, −7.58, and −6.74 kJ mol^{-1} than *L*-Cu-SMOH (−8.75, −4.86, and −4.69 kJ mol^{-1}), primarily engaging residues Tyr16(B), Phe24(B), and the typical FFY segment.

Supplementary references

1 Matsuo, K., Matsushima, Y., Fukuyama, T., Senba, S. & Gekko, K. Vacuum-ultraviolet circular dichroism of amino acids as revealed by synchrotron radiation spectrophotometer. *Chem. Lett.* **31**, 826-827 (2002).

2 Ziegler, M. & von Zelewsky, A. Charge-transfer excited state properties of chiral transition metal coordination compounds studied by chiroptical spectroscopy. *Coord. Chem. Rev.* **177**, 257-300 (1998).

3 Farrar, J. A. *et al.* The electronic structure of CuA: A novel mixed-valence dinuclear copper electron-transfer center. *J. Am. Chem. Soc.* **118**, 11501-11514 (1996).

4 Bravin, C. *et al.* Helicity control of a perfluorinated carbon chain within a chiral supramolecular cage monitored by VCD. *Chem. Commun.* **58**, 2152-2155 (2022).

5 Maeda, K. *et al.* Helix-sense-selective synthesis of right- and left-handed helical luminescent poly(diphenylacetylene)s with memory of the macromolecular helicity and their helical structures. *J. Am. Chem. Soc.* **142**, 7668-7682 (2020).

6 Tamer, Ö. A unique manganese (II) complex of 4-methoxy-pyridine-2-carboxylate: Synthesis, crystal structure, FT-IR and UV–Vis spectra and DFT calculations. *J. Mol. Struct.* **1144**, 370-378 (2017).

7 Teodorescu, F. *et al.* Vibrational circular dichroism of 2,6-di-sec-butyl-4-methylpyridine and 2,6-di-sec-butyl-4-methylpyridine-N-oxide: Theoretical evidence on the existence of multiple –CH, $–CH_2$, and $–CH_3\cdots O$ intramolecular hydrogen bonds on the nitroxide oxygen. *Tetrahedron: Asymmetry* **25**, 725-735 (2014).

8 Bevilacqua, P. C., Kierzek, R., Johnson, K. A. & Turner, D. H. Dynamics of ribozyme binding of substrate revealed by fluorescence-detected stopped-flow methods. *Science* **258**, 1355-1358 (1992).

9 Martial, B., Lefèvre, T., Buffeteau, T. & Auger, M. Vibrational circular dichroism

reveals supramolecular chirality inversion of α-synuclein peptide assemblies upon interactions with anionic membranes. *ACS Nano* **13**, 3232-3242 (2019).

10 Kurouski, D. Advances of vibrational circular dichroism (VCD) in bioanalytical chemistry. A review. *Anal. Chim. Acta* **990**, 54-66 (2017).

11 Kurouski, D., Handen, J. D., Dukor, R. K., Nafie, L. A. & Lednev, I. K. Supramolecular chirality in peptide microcrystals. *Chem. Commun.* **51**, 89-92 (2015).

12 Measey, T. J. & Schweitzer-Stenner, R. Vibrational circular dichroism as a probe of fibrillogenesis: The origin of the anomalous intensity enhancement of amyloid-like fibrils. *J. Am. Chem. Soc.* **133**, 1066-1076 (2011).

13 Kurouski, D., Dukor, Rina K., Lu, X., Nafie, Laurence A. & Lednev, Igor K. Normal and reversed supramolecular chirality of insulin fibrils probed by vibrational circular dichroism at the protofilament level of fibril structure. *Biophys. J.* **103**, 522-531 (2012).

14 Konar, S. *et al.* Structural determination and characterization of copper and zinc bis-glycinates with X-ray crystallography and mass spectrometry. *J. Coord. Chem.* **63**, 3335-3347 (2010).

15 Azeem, M., Li, K., Qin, Y., Dong, L. & Li, W. Mechanical study of a copper dietary supplement, copper glycinate hydrate. *CrystEngComm* **23**, 1815-1820 (2021).

16 Zhang, Y.-Z. *et al.* Interaction of malachite green with bovine serum albumin: Determination of the binding mechanism and binding site by spectroscopic methods. *J. Hazard. Mater.* **163**, 1345-1352 (2009).

17 Ross, P. D. & Subramanian, S. Thermodynamics of protein association reactions: Forces contributing to stability. *Biochemistry* **20**, 3096-3102 (1981).

18 Hou, K. *et al.* Chiral gold nanoparticles enantioselectively rescue memory deficits in a mouse model of Alzheimer's disease. *Nat. Commun.* **11**, 4790 (2020).

19 Gehlen, M. H. The centenary of the Stern-Volmer equation of fluorescence

quenching: from the single line plot to the SV quenching map. *J. Photochem. Photobiol., C* **42**, 100338 (2020).
