# Supplementary material for: Disassembly of chiral hydrogen-bonded frameworks into single-unit organometallic helices for enantioselective amyloid inhibition
Source: Nat Commun. 2025 Aug 27;16:8019. doi: 10.1038/s41467-025-63459-2 (PMC12391419; doi:10.1038/s41467-025-63459-2)
Supplement: Supplementary file 2 — Description of Additional Supplementary Files [file 41467_2025_63459_MOESM2_ESM.pdf]

Description of Additional Supplementary Files

File Name: Supplementary Data 1

Description: CIF of D-Cu-crystal

File Name: Supplementary Data 2

Description: CIF of L-Cu-crystal
