# Supplementary material for: Disassembly of chiral hydrogen-bonded frameworks into single-unit organometallic helices for enantioselective amyloid inhibition
Source: Nat Commun. 2025 Aug 27;16:8019. doi: 10.1038/s41467-025-63459-2 (PMC12391419; doi:10.1038/s41467-025-63459-2)

checkCIF/PLATON report

Structure factors have been supplied for datablock(s) 303-2d

THIS REPORT IS FOR GUIDANCE ONLY. IF USED AS PART OF A REVIEW PROCEDURE FOR PUBLICATION, IT SHOULD NOT REPLACE THE EXPERTISE OF AN EXPERIENCED CRYSTALLOGRAPHIC REFEREE.

No syntax errors found. CIF dictionary Interpreting this report

Datablock: 303-2d

```
Bond precision:   C-C = 0.0046 A          Wavelength=1.54184

Cell:              a=9.1216(1)      b=9.1216(1)      c=35.5635(3)
                   alpha=90         beta=90          gamma=90
Temperature:       100 K
```

	Calculated	Reported
Volume	2959.01(7)	2959.01(7)
Space group	P 43 21 2	P 43 21 2
Hall group	P 4nw 2abw	P 4nw 2abw
Moiety formula	C10 H15 Cl Cu N2 O4 [+ solvent]	C10 H15 Cl Cu N2 O4
Sum formula	C10 H15 Cl Cu N2 O4 [+ solvent]	C10 H15 Cl Cu N2 O4
Mr	326.24	326.23
Dx,g cm-3	1.465	1.465
Z	8	8
Mu (mm-1)	3.834	3.834
F000	1336.0	1336.0
F000'	1326.49	
h,k,lmax	11,11,44	11,10,43
Nref	3020[1856]	2947
Tmin,Tmax	0.614,0.682	0.543,1.000
Tmin'	0.557	

```
Correction method= # Reported T Limits: Tmin=0.543 Tmax=1.000
AbsCorr = MULTI-SCAN

Data completeness= 1.59/0.98        Theta(max)= 74.374
```

R(reflections)= 0.0294(2897)

wR2(reflections)= 0.0736(2947)

S = 1.075 Npar= 166

The following ALERTS were generated. Each ALERT has the format
test-name_ALERT_alert-type_alert-level.
Click on the hyperlinks for more details of the test.

Alert level C

```
PLAT420_ALERT_2_C D-H Bond Without Acceptor  N2       --H2       .     Please Check
PLAT911_ALERT_3_C Missing FCF Refl Between Thmin & STh/L=    0.600          2 Report
                0 10  0,   2  2  3,
```

Alert level G

```
PLAT004_ALERT_5_G Polymeric Structure Found with Maximum Dimension          1 Info
PLAT007_ALERT_5_G Number of Unrefined Donor-H Atoms .............           4 Report
              H1    H2    H4A   H4B
PLAT143_ALERT_4_G s.u. on c - Axis Small or Missing .............     0.00030 Ang.
PLAT605_ALERT_4_G Largest Solvent Accessible VOID in the Structure        118 A**3
PLAT794_ALERT_5_G Tentative Bond Valency for Cu1       (II)      .       2.10 Info
PLAT868_ALERT_4_G ALERTS Due to the Use of _smtbx_masks Suppressed          ! Info
PLAT912_ALERT_4_G Missing # of FCF Reflections Above STh/L=  0.600         16 Note
PLAT933_ALERT_2_G Number of HKL-OMIT Records in Embedded .res File          1 Note
                2  2  3,
PLAT969_ALERT_5_G The 'Henn et al.' R-Factor-gap value ..........        3.59 Note
              Predicted wR2: Based on SigI**2  2.05 or SHELX Weight  7.05
PLAT978_ALERT_2_G Number C-C Bonds with Positive Residual Density.          1 Info
```

---

   0 **ALERT level A** = Most likely a serious problem - resolve or explain
   0 **ALERT level B** = A potentially serious problem, consider carefully
   2 **ALERT level C** = Check. Ensure it is not caused by an omission or oversight
  10 **ALERT level G** = General information/check it is not something unexpected

   0 ALERT type 1 CIF construction/syntax error, inconsistent or missing data
   3 ALERT type 2 Indicator that the structure model may be wrong or deficient
   1 ALERT type 3 Indicator that the structure quality may be low
   4 ALERT type 4 Improvement, methodology, query or suggestion
   4 ALERT type 5 Informative message, check

It is advisable to attempt to resolve as many as possible of the alerts in all categories. Often the minor alerts point to easily fixed oversights, errors and omissions in your CIF or refinement strategy, so attention to these fine details can be worthwhile. In order to resolve some of the more serious problems it may be necessary to carry out additional measurements or structure refinements. However, the purpose of your study may justify the reported deviations and the more serious of these should normally be commented upon in the discussion or experimental section of a paper or in the "special_details" fields of the CIF. checkCIF was carefully designed to identify outliers and unusual parameters, but every test has its limitations and alerts that are not important in a particular case may appear. Conversely, the absence of alerts does not guarantee there are no aspects of the results needing attention. It is up to the individual to critically assess their own results and, if necessary, seek expert advice.

Publication of your CIF in IUCr journals

A basic structural check has been run on your CIF. These basic checks will be run on all CIFs submitted for publication in IUCr journals (*Acta Crystallographica*, *Journal of Applied Crystallography*, *Journal of Synchrotron Radiation*); however, if you intend to submit to *Acta Crystallographica Section C* or *E* or *IUCrData*, you should make sure that full publication checks are run on the final version of your CIF prior to submission.

Publication of your CIF in other journals

Please refer to the *Notes for Authors* of the relevant journal for any special instructions relating to CIF submission.

PLATON version of 13/12/2023; check.def file version of 13/12/2023

Datablock 303-2d - ellipsoid plot

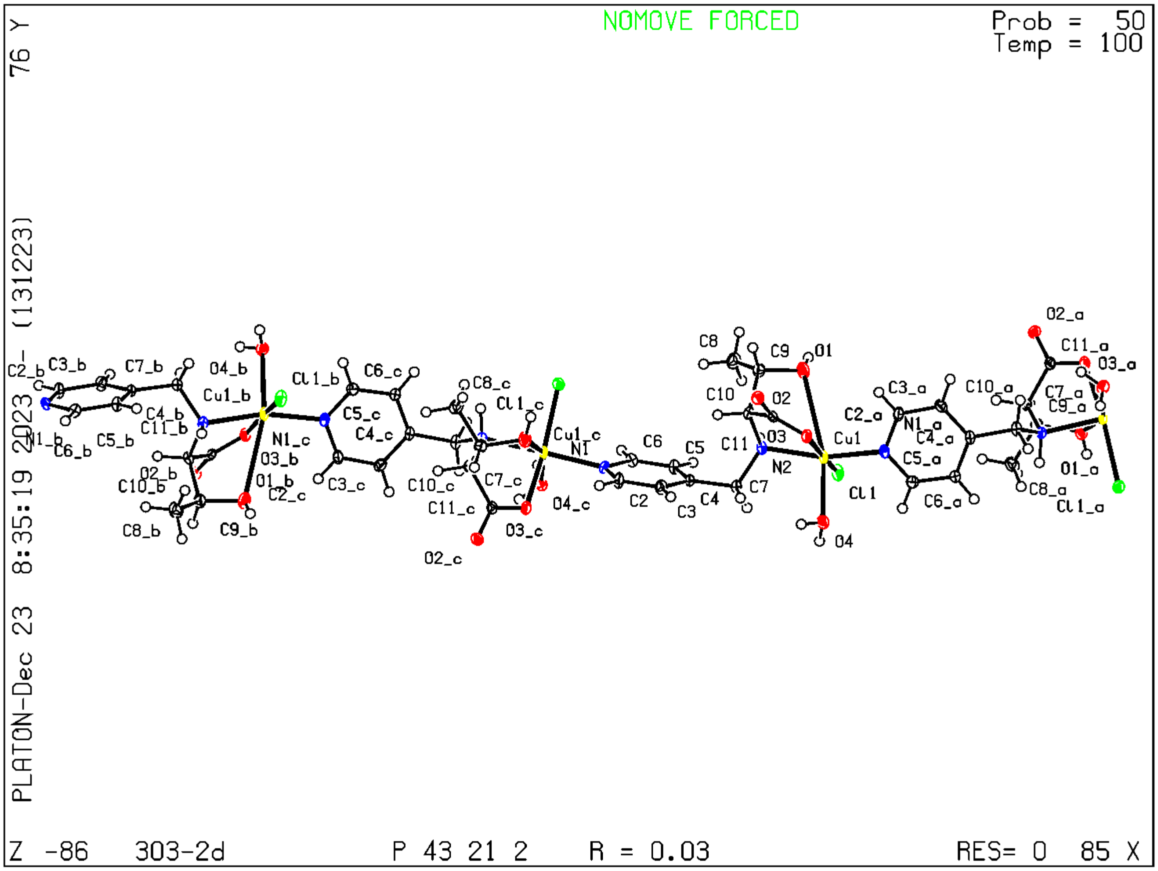

Supplement: Supplementary file 3 — Supplementary Data 1 [file 41467_2025_63459_MOESM3_ESM.zip › 303-2d.pdf]
