# Supplementary material for: Disassembly of chiral hydrogen-bonded frameworks into single-unit organometallic helices for enantioselective amyloid inhibition
Source: Nat Commun. 2025 Aug 27;16:8019. doi: 10.1038/s41467-025-63459-2 (PMC12391419; doi:10.1038/s41467-025-63459-2)
Supplement: Supplementary file 5 — Reporting Summary [file 41467_2025_63459_MOESM5_ESM.pdf]

nature portfolio

Corresponding author(s): Jun Guo

Last updated by author(s): Jul 31, 2025

Reporting Summary

Nature Portfolio wishes to improve the reproducibility of the work that we publish. This form provides structure for consistency and transparency in reporting. For further information on Nature Portfolio policies, see our Editorial Policies and the Editorial Policy Checklist.

Statistics

For all statistical analyses, confirm that the following items are present in the figure legend, table legend, main text, or Methods section.

n/a	Confirmed	
☐	☒	The exact sample size (*n*) for each experimental group/condition, given as a discrete number and unit of measurement
☒	☐	A statement on whether measurements were taken from distinct samples or whether the same sample was measured repeatedly
☐	☒	The statistical test(s) used AND whether they are one- or two-sided<br>*Only common tests should be described solely by name; describe more complex techniques in the Methods section.*
☒	☐	A description of all covariates tested
☐	☒	A description of any assumptions or corrections, such as tests of normality and adjustment for multiple comparisons
☐	☒	A full description of the statistical parameters including central tendency (e.g. means) or other basic estimates (e.g. regression coefficient) AND variation (e.g. standard deviation) or associated estimates of uncertainty (e.g. confidence intervals)
☒	☐	For null hypothesis testing, the test statistic (e.g. *F*, *t*, *r*) with confidence intervals, effect sizes, degrees of freedom and *P* value noted<br>*Give P values as exact values whenever suitable.*
☒	☐	For Bayesian analysis, information on the choice of priors and Markov chain Monte Carlo settings
☒	☐	For hierarchical and complex designs, identification of the appropriate level for tests and full reporting of outcomes
☒	☐	Estimates of effect sizes (e.g. Cohen's *d*, Pearson's *r*), indicating how they were calculated

Our web collection on statistics for biologists contains articles on many of the points above.

Software and code

Policy information about availability of computer code

Data collection	Circular dichroism spectrum data was acquired using Chirascan 4.8.3.0.<br>Isothermal titration calorimeter data was acquired using ITCRun ver 3.7.<br>Fluorescence data was collected using the ZWin fluorescence spectrophotometer 1.0.
Data analysis	Crystallographic information was made using VESTA and Diamond.<br>Circular dichroism spectrum data was processed using Pro-Data Viewer software.<br>Isothermal titration calorimeter data was fitted using Launch NanoAnalyze software.<br>All analyses of the data appearing in the manuscript were performed using Origin 2021.

For manuscripts utilizing custom algorithms or software that are central to the research but not yet described in published literature, software must be made available to editors and reviewers. We strongly encourage code deposition in a community repository (e.g. GitHub). See the Nature Portfolio guidelines for submitting code & software for further information.

Data

Policy information about availability of data

All manuscripts must include a data availability statement. This statement should provide the following information, where applicable:

- Accession codes, unique identifiers, or web links for publicly available datasets
- A description of any restrictions on data availability
- For clinical datasets or third party data, please ensure that the statement adheres to our policy

The authors declare that all data supporting the findings of this study are available within the paper and its supplementary information files. Source data are provided with this paper. Crystallographic data have been deposited at the Cambridge Crystallographic Data Centre with deposition numbers of CCDC 2330456 and 2330457.

Research involving human participants, their data, or biological material

Policy information about studies with human participants or human data. See also policy information about sex, gender (identity/presentation), and sexual orientation and race, ethnicity and racism.

Reporting on sex and gender	Our research is not involved in human research.
Reporting on race, ethnicity, or other socially relevant groupings	Our research is not involved in human research.
Population characteristics	Our research is not involved in human research.
Recruitment	Our research is not involved in human research.
Ethics oversight	Our research is not involved in human research.

Note that full information on the approval of the study protocol must also be provided in the manuscript.

Field-specific reporting

Please select the one below that is the best fit for your research. If you are not sure, read the appropriate sections before making your selection.

☒ Life sciences ☐ Behavioural & social sciences ☐ Ecological, evolutionary & environmental sciences

For a reference copy of the document with all sections, see nature.com/documents/nr-reporting-summary-flat.pdf

Life sciences study design

All studies must disclose on these points even when the disclosure is negative.

Sample size	We tested 3 parallel samples each time.
Data exclusions	No data were excluded from the analyses.
Replication	Independent replication was done.
Randomization	Our research is not involved in clinical Trial.
Blinding	Our research is not involved in clinical Trial.

Reporting for specific materials, systems and methods

We require information from authors about some types of materials, experimental systems and methods used in many studies. Here, indicate whether each material, system or method listed is relevant to your study. If you are not sure if a list item applies to your research, read the appropriate section before selecting a response.

Materials & experimental systems

n/a	Involved in the study
☒	☐ Antibodies
☒	☐ Eukaryotic cell lines
☒	☐ Palaeontology and archaeology
☒	☐ Animals and other organisms
☒	☐ Clinical data
☒	☐ Dual use research of concern
☒	☐ Plants

Methods

n/a	Involved in the study
☒	☐ ChIP-seq
☒	☐ Flow cytometry
☒	☐ MRI-based neuroimaging

Plants

Seed stocks: Our research is not involved with any plants.

Novel plant genotypes: Our research is not involved with any plants.

Authentication: Our research is not involved with any plants.
